# Structure of human cytoplasmic Pol II complex explains global transcription repression by GDOWN1

**DOI:** 10.1038/s41467-026-76334-5

**Published:** 2026-08-05

**Authors:** Jana Schmitzová, Yumeng Zhan, Srinivasan Rengachari, Frauke Grabbe, Olexandr Dybkov, Henning Urlaub, Michael Lidschreiber, Christian Dienemann, Patrick Cramer

**Affiliations:** 1 Department of Molecular Biology, Max Planck Institute for Multidisciplinary Sciences, Göttingen, Germany; 2 Research Group Bioanalytical Mass Spectrometry, Max Planck Institute for Multidisciplinary Sciences, Göttingen, Germany; 3 Bioanalytics, Department of Clinical Chemistry, University Medical Center, Göttingen, Germany; 4 Present Address: Section of Structural and Synthetic Biology, Department of Infectious Disease, Imperial College London, London, UK

**Keywords:** Cryoelectron microscopy, Cryoelectron microscopy, Transcription

## Abstract

RNA polymerase II (Pol II) is a 12-subunit enzyme crucial for gene transcription in the nucleus. However, its assembly in the cytoplasm, nuclear import, and nuclear function of assembly factors remain poorly understood. Here, we isolated Pol II from the cytoplasmic fraction of human cells (cfPol II) and find it associated with the assembly and transport factors GDOWN1, RPAP2, GPN1, and GPN3. Cryo-EM analysis of cfPol II resolves RPAP2 and GDOWN1 bound to Pol II at 2.9 Å resolution and shows that Pol II is fully assembled in the cytoplasm before nuclear import. Our structure of GDOWN1 bound to the Pol II surface reveals three distinct regions of GDOWN1 that interact with the RPB2 protrusion domain, RPB3, and RPB10. Biochemical analyses show that GDOWN1 facilitates soluble expression of a subcomplex comprising RPB3, RPB10, RPB11 and RPB12, suggesting a role for GDOWN1 in Pol II assembly. Further, GDOWN1 binding to Pol II overlaps with binding sites of the essential transcription factors IIB and IIF, rendering cfPol II inactive in promoter-dependent transcription initiation in vitro. Our results provide a basis for GDOWN1-dependent global transcription repression and suggest a model for a role of GDOWN1 in Pol II assembly, import, and transcription regulation.

## Abstract (web summary)

The assembly and nuclear import of RNA polymerase II (Pol II) are not fully understood. Here, the authors identify GDOWN1’s role in Pol II assembly and transcription regulation using biochemical and cryo-EM analyses of cytoplasmic Pol II.

## Introduction

RNA polymerase II (Pol II) is a 12-subunit complex that is responsible for transcription of protein-coding genes and various regulatory RNAs, including snRNAs, lncRNAs, miRNAs, and eRNAs across eukaryotes^1^. Pol II function is essential for cell differentiation, development, and environmental responses^2^. The first high-resolution structure of a eukaryotic Pol II was determined over two decades ago^3^ and since then, a large amount of structural and mechanistic data have accumulated. However, the majority of these studies have focused on understanding how Pol II regulates gene expression in the nucleus^4^. On the other hand, the molecular basis underlying Pol II assembly in the cytoplasm, its nuclear import, and the intranuclear release of assembly and transport factors from Pol II remain less well known.

Studies in yeast and subsequent work in human systems have shed light on some key aspects of Pol II biogenesis and assembly^5–8^. Pol II assembly proceeds via two major intermediates centered on its largest subunits—RPB1 and RPB2. First, the formation of an RPB2-containing subcomplex comprising the Pol II subunits RPB2, RPB9, RPB3, RPB11, RPB10, and RPB12 is assisted by the GTPases GPN1, GPN2, GPN3, RNA polymerase II associated proteins 1 and 2 (RPAP1, RPAP2), and GDOWN1^9–13^. Second, the RPB1-containing subassembly, consisting of RPB1, RPB5, RPB6, and RPB8, is assembled and stabilized by heat-shock protein 90 (HSP90) and the R2TP complex^14^. These subassemblies then consolidate with the RPB4/RPB7 heterodimer to constitute the complete 12-subunit Pol II complex.

We still lack an understanding of the exact stoichiometry of Pol II subunits during nuclear import. However, depletion of individual Pol II subunits results in cytoplasmic accumulation of RPB1, suggesting that the complete assembly of Pol II precedes its nuclear import^7,14^. SLC7A6OS (yeast Iwr1^15^), which likely binds a fully assembled Pol II, facilitates nuclear import of Pol II. Assembly factors that are still associated with Pol II are imported together with Pol II^5,11,12,16,17^.

Among the factors needed for Pol II assembly, GDOWN1, GPN1, GPN3, and RPAP2 are the most characterized. GDOWN1 is a metazoan-specific protein that resides predominantly in the cytoplasm, either in its free form, as part of an RPB2-containing Pol II subassembly, or bound to fully assembled Pol II^7^. GDOWN1 is imported to the nucleus together with Pol II^18^. In addition, GDOWN1 has been shown to interfere with Pol II transcription in the nucleus at the level of initiation^19–23^, pausing^20^, elongation^20,24^ as well as termination^25^. Additionally, GDOWN1 accumulates in the nucleus at the onset of mitosis, where it was proposed to act as a global suppressor of Pol II transcription^18^. GPN1 and GPN3 form a complex that can interact with the CTD of RPB1, and with RPB4/RPB7^11^. The GTPase activity of GPN1 is required for Pol II nuclear localization, and both GPN1 and GPN3 are essential for cell viability, underscoring their critical role in Pol II assembly^3,11^. RPAP2 (yeast Rtr1^26^) shuttles between the cytoplasm and nucleus in a GPN1-dependent manner^17^. RPAP2 controls gene expression by modulating Pol II transcriptional activity and regulating nuclear Pol II levels^17^.

To date, structural insights into Pol II regulation by assembly and import factors are limited to high-resolution structures of Pol II-RPAP2 complexes^27,28^, low-resolution models of GDOWN1 bound to Pol II^29,30^ in humans, and a yeast Pol II-Iwr1 complex^15^. Structures of Pol II in complex with more than one assembly factor are lacking, which limits deeper mechanistic understanding of these factors in Pol II assembly, import, and their associated functions after import to the nucleus.

To address this, we have developed a system for the isolation of human Pol II from the cytoplasmic fraction (cfPol II) of HEK293 cells expressing N-terminally tagged RPB1. Using biochemical and proteomic characterization of cfPol II, we confirmed the co-purification and binding of the well-known Pol II assembly and import factors, including GDOWN1, GPN1, GPN3, and RPAP2. Single-particle cryo-EM analysis of cfPol II reveals a high-resolution structure of human Pol II bound by two assembly factors, namely RPAP2 and GDOWN1. The structure shows how GDOWN1 binds to the Pol II surface in a multi-valent manner. Co-expression experiments indicate that GDOWN1 facilitates expression of an RPB3-containing Pol II subassembly. Comparative structural analysis explains the incompatibility between GDOWN1 and transcription initiation factors. Further, we show that cfPol II is inactive in de novo transcription initiation in vitro, and the inhibition is mainly caused by GDOWN1. These findings explain how GDOWN1 can globally repress transcription and suggest a model for the cellular roles of GDOWN1.

## Results

### Isolation and cryo-EM studies of human cfPol II

**Fig. 1:**
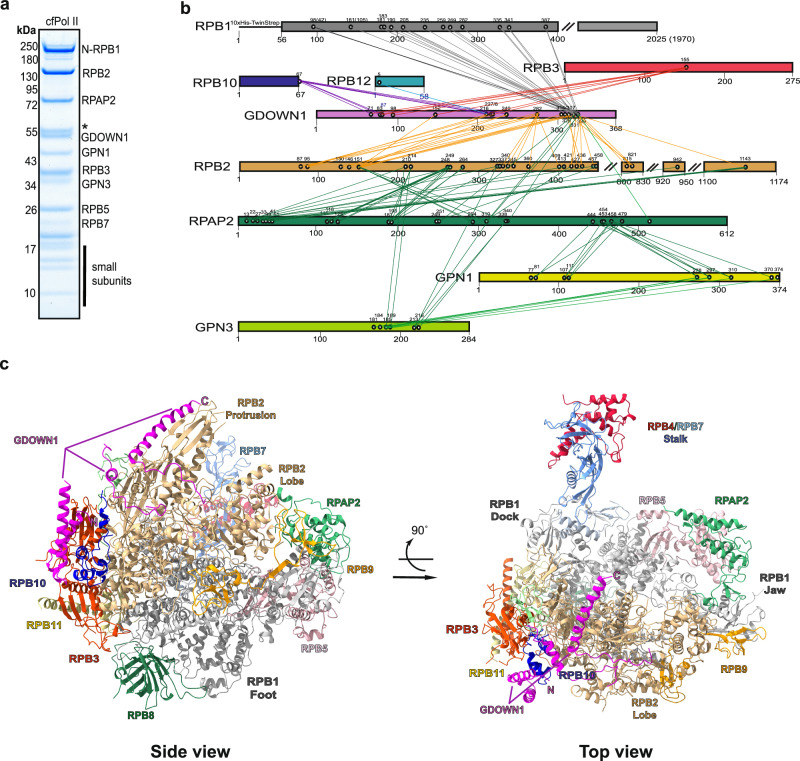
Structure of the human Pol II–RPAP2–GDOWN1 complex. **a** SDS-PAGE analysis of purified endogenous human Pol II from the cytoplasmic fraction (cfPol II) by Size-exclusion chromatography. The purification of cfPol II was performed using different batches of cytoplasmic fractions from HEK293 cells stably expressing N-terminally His10-Twin-Strep-tagged human Pol II subunit RPB1, and this process was repeated at least four times. *Left:* Molecular weight marker (kDa). *Right:* Names of the Pol II subunits and the assembly factors as identified by mass spectrometry. **b** Intermolecular crosslinking network of purified endogenous cfPol II. Diagram shows crosslinks between: GDOWN1 and Pol II subunits (RPB1, RPB2, RPB3, RPB10, RPB12); RPAP2 and RPB2; RPAP2 and GPN1/GPN3; and RPB2 and GPN3. Color coding is consistent with Cramer et al.^3^. **c** Ribbon representations of the complex. *Left:* Side view of human Pol II highlighting the GDOWN1 interaction platform (magenta). *Right:* Top view of human Pol II detailing the RPAP2–Pol II interface (green). Color coding of Pol II subunits is consistent with Cramer et al.^3^. Source data are provided as a Source data file.

To investigate cfPol II, we purified Pol II complexes from the cytoplasmic fraction of HEK293 cells expressing Pol II that is tagged at the RBP1 N-terminus^31^ (“Methods”, Supplementary Fig. 1a, b). Biochemical analysis of cfPol II reveals that it is bound by several additional proteins (Fig. 1a). Chemical crosslinking coupled to mass spectrometry (XL-MS, Supplementary Fig. 1c) showed that cfPol II comprises all subunits of Pol II as well as GPN1, GPN3, RPAP2, and GDOWN1 (Fig. 1b, Supplementary Table 1, Supplementary Data 1 and 2). The composition of cfPol II is consistent with the current model for Pol II assembly in the cytoplasm and subsequent nuclear import^5,7^. Initial cryo-EM analysis of this sample resulted in a reconstruction with clear density for only Pol II and RPAP2 (data not shown). To improve the homogeneity and stoichiometry of cfPol II for cryo-EM studies, we added recombinantly purified GDOWN1, RPAP2, GPN1, and GPN3 and purified the complex by size exclusion chromatography (Supplementary Fig. 1d). This complex was mildly crosslinked to improve its stability. Subsequent XL-MS analysis showed that the complex composition is highly similar to the composition of initially purified cfPol II (Supplementary Fig. 1d, Supplementary Table 1, Supplementary Data 1 and 2). We then subjected this cfPol II sample to single-particle cryo-EM for structural analysis (Methods).

Analysis of the cryo-EM data (Supplementary Figs. 2 and 3) yielded a cfPol II reconstruction at an overall resolution of 2.9 Å (Map A) and 2.6 Å (Map B) (Fig. 1c, Supplementary Fig. 4, Supplementary Table 2). Most regions of Pol II are well resolved except for the clamp domain, which remains largely absent despite attempts to resolve it by focused classification (Supplementary Fig. 5a). This phenomenon was observed before for free Pol II^32^ as well as for the Pol II-RPAP2 complexes^27,28^. Consistent with the composition of cfPol II (Fig. 1a), density for the conserved N-terminus of RPAP2 bound to RPB5 and the RPB1 jaw domain is well resolved (Supplementary Fig. 5a). RPAP2 positioning in our structure is largely identical to previously determined Pol II-RPAP2 complex structures, with minor deviations^27,28^ (Supplementary Fig. 5b–d). We did not identify any corresponding density for GPN1 and GPN3, although biochemical and XL-MS analyses confirmed their presence in the endogenous cfPol II sample as well as in the sample used for cryo-EM (Supplementary Fig. 1c–e; Fig. 1b), and both are known to interact with RPAP2 and the Pol II CTD and stalk^11^. This suggests a weaker association or structural flexibility of GPN1 and GPN3 relative to cfPol II. After unambiguously fitting Pol II and RPAP2, unassigned cryo-EM density was present at the Pol II lobe and protrusion domains (Supplementary Fig. 6a). Previous low-resolution cryo-EM structures for GDOWN1 bound to Pol II have localized GDOWN1 to the Pol II lobe and protrusion regions (Supplementary Fig. 6b)^30^. We therefore carefully sorted and locally refined our cryo-EM data to resolve this density to a local resolution range of 2.9–4.2 Å (Supplementary Fig. 2). Using this improved cryo-EM map and an AlphaFold 3^33^ predicted model of a Pol II-RPAP2-GDOWN1 complex (Supplementary Fig. 6d), we unambiguously assigned and built GDOWN1 residues 19–73, 224–255, and 290–334 into the cfPol II structure (Supplementary Figs. 6e and 7). In summary, our purification strategy enabled the preparation of a stochiometric cfPol II complex comprising RPAP2, GDOWN1, GPN1, and GPN3 bound to Pol II. Cryo-EM analysis of this sample led to a high-resolution structural model of the Pol II-RPAP2-GDOWN1 complex.

### Structure of GDOWN1 bound to Pol II

**Fig. 2:**
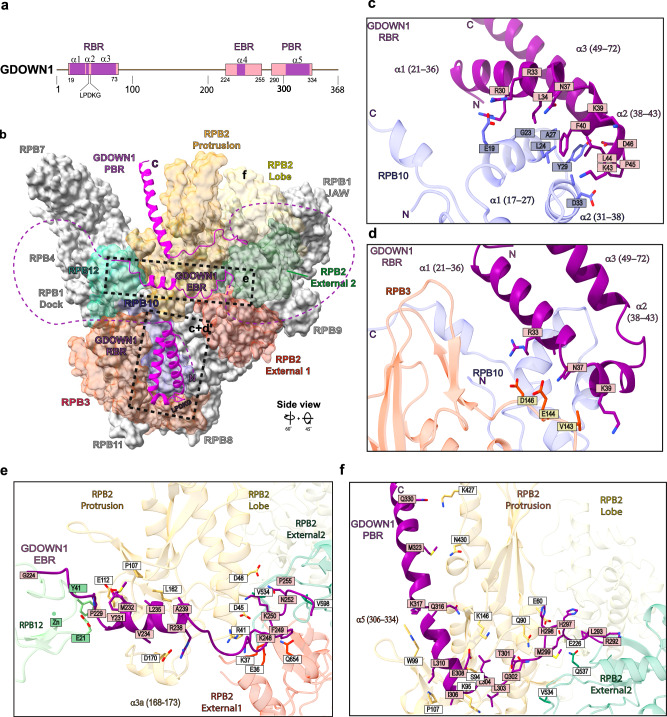
GDOWN1 binding interfaces with Pol II subunits. **a** Domain architecture of human GDOWN1. Modeled regions are shown in magenta: N-terminus (residues 19–73), named as RPB10 Binding Region (RBR); middle region (residues 224–255), named as External Binding Region (EBR); C-terminus (residues 290–334), named as Protrusion Binding Region (PBR). The magenta line indicates disordered regions unresolved in cryo-EM Map A. **b** Surface representation of human cfPol II, highlighting GDOWN1-binding sites on Pol II. The ribbon model of human Pol II (initial side view orientation) was rotated 60° around the y-axis, followed by a 45° rotation around the *x*-axis. Domains of RPB2, proteins RPB10, RPB3, and RPB12 follow consistent coloring throughout, as in Cramer et al.^3^. GDOWN1 is shown as a magenta ribbon with unstructured regions marked by magenta dashed lines. **c** Interaction interface between GDOWN1 RBR (residues 19–73) and RPB10. **d** Interaction interface between GDOWN1 RBR (residues 19–73) and RPB3. **e** Interaction interface between GDOWN1 EBR (residues 224–255) and RPB2 (protrusion, external 1 and 2 domains). **f** Interaction interface between GDOWN1 PBR (residues 290–334) and RPB2 (protrusion domain, external domain 2). Color coding matches (**b**).

Our high-resolution cryo-EM structure of the Pol II–RPAP2–GDOWN1 complex provides an atomic model of GDOWN1. We identified three distinct regions of GDOWN1 that bind to Pol II (Fig. 2a, b). First, the N-terminal part of GDOWN1 (residues 19–73) binds to Pol II subunit RPB10, which we refer to as the RPB10-binding region (RBR, Fig. 2a, b). The RBR comprises three α-helices (α1–α3) and includes the conserved LPDKG motif^25^. It binds primarily to RPB10 helices α1 and α2 via hydrophobic and polar interactions (Fig. 2c), with additional polar and electrostatic contacts between GDOWN1 α1 and RPB3 (Fig. 2d). Notably, residues R30, R33, and L44 contribute to the Pol II binding interface, consistent with previous biochemical studies^25,30^. Second, the central part of GDOWN1 resolved in our structure (residues 224–255) binds to the Pol II protrusion and external 1 and 2 domains. We refer to this region as the external-binding region (EBR, Fig. 2a, b). The EBR contains a single α-helix (α4) flanked by unstructured segments. GDOWN1 α4 forms hydrophobic contacts with RPB2 helix α3 and RPB12, while the surrounding flexible regions form a mix of hydrophobic, polar, and charged interactions with the protrusion, external and lobe domains of RPB2 (Fig. 2e). Third, the C-terminal part of GDOWN1 (residues 290–344) binds to the Pol II protrusion domain and we name it protrusion binding domain (PBR, Fig. 2a, b). The α5 (residues 305–344) of the PBR anchors GDOWN1 to the tip of the protrusion while the less structured stretches bind to the protrusion base (Fig. 2f). Residues L303 and L304, previously shown to be critical for transcription inhibition^30^, participate in a hydrophobic interaction network at the protrusion base.

The positioning of the RBR, EBR, and PBR in our Pol II -RPAP2-GDOWN1 structure shows significant differences from the previous work^30^ (Supplementary Fig. 6b, c). In particular, the RBR comprises residues 19–73, whereas the N-terminal transcription inhibition region (N-TIR) was proposed to range from 1 to 65. Next, the residue range of the EBR (224–255) differs from the Pol II binding region I (216–299) that was suggested to mediate Pol II binding. Finally, the RBR (residues 290–334) comprises the previously identified C-terminal transcription inhibition region (C-TIR) and Pol II binding region II; however, our structure shows that they rather belong to a single region. Altogether, our high-resolution cryo-EM structure of GDOWN1 allowed us to build an atomic model for GDOWN1 bound to the Pol II surface. We resolved three distinct structural regions of GDOWN1 that bind to Pol II in a multi-valent manner.

### GDOWN1 facilitates the expression of an RPB3 subcomplex

Our structure reveals that GDOWN1 binds to Pol II subunits RPB2, RPB3, RPB10 and RPB12, and bridges between Pol II subunits RPB2 and RPB3 (Fig. 2). Based on similarity to the assembly of bacterial RNA polymerase, RPB2 and RPB3 have been hypothesized to be part of separate subcomplexes that then associate to form the RPB2, RPB9, RPB3, RPB10, RPB11 and RPB12 subassembly at an early step of Pol II assembly^7^. To investigate whether GDOWN1 facilitates the formation of the RPB3 subcomplex, which includes RPB3, RPB10, RPB11, and RPB12, we expressed these subunits in insect cells with and without co-expression of GDOWN1 (Supplementary Fig. 8a). We then used affinity purification with amylose beads to monitor expression of the subcomplex components by SDS-PAGE. When expressing 6xHis-MBP-RPB3, RPB10, RPB11, and RPB12, we could only pull down RPB11, while RPB10 and RPB12 were not pulled down. In contrast, co-expression of 6xHis-MBP-GDOWN1 allowed us to purify RPB3, RPB10, RPB11, and RPB12. We further tested co-expression of RPB2 and RPB9 with and without GDOWN1 (Supplementary Fig. 8b). While both subunits were expressed without GDOWN1, the inclusion of GDOWN1 led to a loss of RPB9. This suggests that GDOWN1 is not required to facilitate the interaction between RPB2 and RPB9, which aligns with the absence of clear interactions between GDOWN1 and RPB9 in our structure. Moreover, our data indicate that GDOWN1 may interfere with the association of RPB9 to RPB2 during Pol II assembly.

In summary, our co-expression and affinity purification studies demonstrate that GDOWN1 facilitates the expression of the RPB3 subcomplex, comprising RPB3, RPB10, RPB11, and RPB12. However, the expression of the RPB2-RPB9 subcomplex does not benefit from the presence of GDOWN1 and likely requires other assembly factors for stabilization and joining the RPB3 subcomplex.

### GDOWN1 binding to Pol II overlaps with transcription initiation factors

**Fig. 3:**
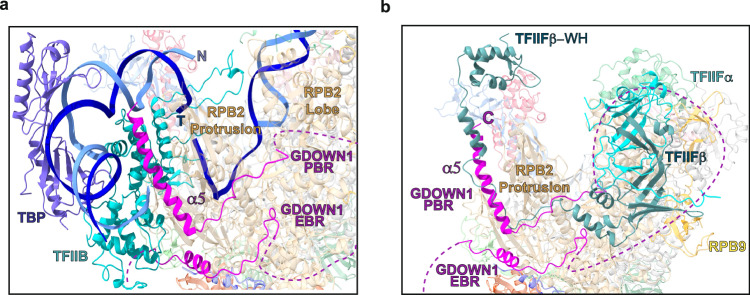
GDOWN1 binding to Pol II sterically clashes with transcription initiation machinery. **a** Structural clash analysis. GDOWN1 PBR (magenta ribbon) overlaps with transcription initiation factors TBP (blue) and TFIIB (cyan), and promoter DNA. A Pol II pre-initiation complex (PIC) model (PDB: 8S52^34^) was superimposed on our Pol II-RPAP2-GDOWN1 structure. Pol II is shown at 80% transparency. Color scheme matches Fig. 1. **b** Structural clash analysis. GDOWN1 PBR (magenta ribbon) overlaps with the linker between the dimerization domain and the C-terminal WH domain of TFIIF-β, and the region between the PBR and EBR might sterically interfere with the binding of TFIIF to the Pol II lobe. Color scheme matches Fig. 1.

Multiple studies have shown that GDOWN1 competes with transcription initiation^19,20,30^ factors to bind Pol II, thereby interfering with transcription in the nucleus^21,22^. To explore the structural basis underlying these phenomena, we compared our high-resolution model of GDOWN1 bound to Pol II to the structure of the pre-initiation complex (PIC)^34^. The comparison identifies three main clashes between GDOWN1 and general transcription factors (GTFs). First, the GDOWN1 PBR overlaps with the TFIIB cyclin domains (Fig. 3a). Second, the α5-helix of the PBR extends into the trajectory of promoter DNA (Fig. 3a). Third, the path of the PBR on the Pol II surface largely overlaps with the linker between the dimerization domain and the C-terminal WH domain of TFIIF-β (Fig. 3b). Moreover, although the region between the PBR and EBR is disordered, it might sterically interfere with binding of TFIIF to the Pol II lobe (Fig. 3b). These clashes are consistent with studies showing that GDOWN1 competes with TFIIB and TFIIF in the PIC and inhibits transcription initiation^19,20,22,30^. Altogether, our structure of the Pol II-RPAP2-GDOWN1 complex shows how GDOWN1 binding to Pol II may preclude transcription initiation factors from binding Pol II and thus explains how GDOWN1 can inhibit PIC assembly and transcription initiation.

### cfPol II containing GDOWN1 and RPAP2 is transcriptionally inactive

**Fig. 4:**
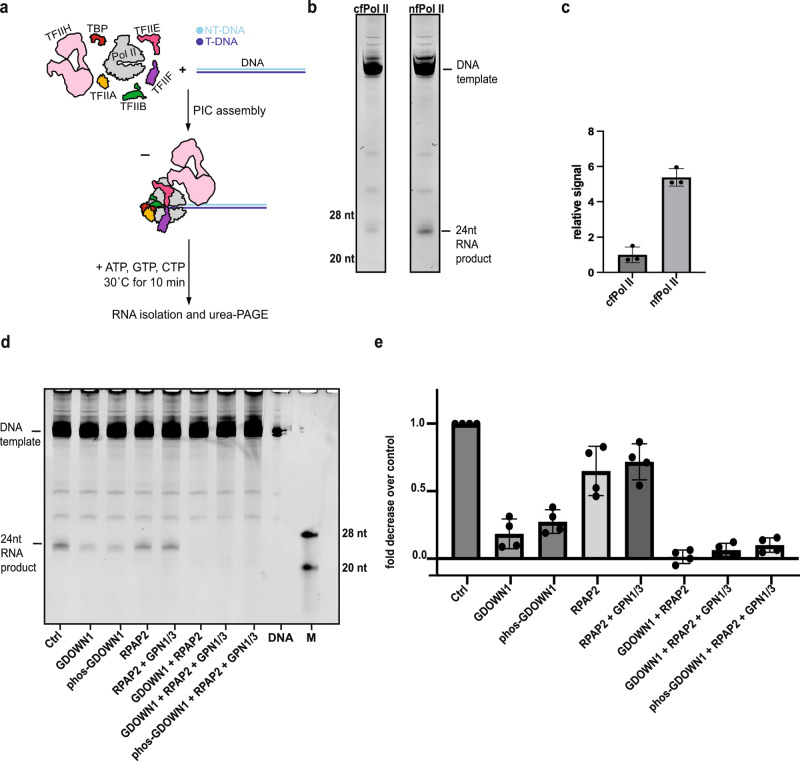
Purified cfPol II containing GDOWN1 and RPAP2 is transcriptionally inactive. **a** Schematic of the de novo transcription initiation assay performed in vitro. **b** Biochemical validation. 20% Urea-PAGE analysis of transcription products. *Left:* Transcription initiation assay performed with purified endogenous cytoplasmic Pol II (cfPol II). *Right:* Transcription initiation assay performed with purified endogenous nuclear Pol II (nfPol II). 28 nt and 20 nt bands are RNA markers. DNA: Template DNA used in the transcription initiation assay. Experiments performed in technical replicates (*n* = 3). Nuclear Pol II was isolated as previously described^31^; cytoplasmic Pol II was prepared as in Supplementary Fig. 1a and “Methods”. **c** Quantification of the RNA products from (**b**) (Methods). Mean ± standard deviation (SD) values are indicated. **d** Analysis of the effect of different assembly factors present in purified cfPol II (GDOWN1, RPAP2, GPN1, GPN3) on transcription initiation. Assembly factors were added in 5x access to TFIIF. The transcription products were analyzed using 20% Urea-PAGE. M: RNA marker (28 nt and 20 nt bands). DNA: Template DNA used in the transcription initiation assay. Experiments performed in technical replicates (*n* = 4). Nuclear Pol II was isolated as previously described^31^. **e** Quantification of the RNA products from (**d**) (Methods). Mean ± standard deviation (SD) values are indicated. Source data are provided as a Source data file.

The structural incompatibility between GDOWN1 and parts of the Pol II PIC indicates that cfPol II may be unable to initiate transcription. Additionally, RPAP2 was shown to have a mild inhibitory effect on transcription initiation by Pol II^28^. To test whether cfPol II is able to initiate transcription in vitro, we performed transcription initiation assays using promoter DNA and purified GTFs (“Methods”, Fig. 4a). To monitor transcription initiation while reducing the influence of altered transcription elongation, transcription reactions were performed using promoter DNA containing a 24 bp U-less cassette.

When purified cfPol II was used in the transcription initiation reaction, only a weak signal for the expected 24 nt RNA product could be detected (Fig. 4b, c). In contrast, Pol II that was purified from the nuclear fraction (nfPol II) from the same cell line initiates transcription de novo, and RNA synthesis was observed (Fig. 4b, c). We next asked which assembly factor present in purified cfPol II contributes to the inhibition of transcription initiation. To test this, we added recombinantly expressed and purified GDOWN1, RPAP2, GPN1, and GPN3 in fivefold excess over the amount of TFIIF (Supplementary Fig. 9) to nfPol II and performed transcription initiation assays as described for cfPol II and nfPol II (Fig. 4a). The addition of RPAP2 alone reduces transcription by ~40%, which is in a similar range to what has been previously reported for RPAP2^28^ (Fig. 4d, e). RPAP2 in complex with GPN1 and GPN3 inhibits transcription by 30%, a reduction similar to that observed with RPAP2 alone (Fig. 4d, e), suggesting that GPN1 and GPN3 do not interfere with transcription initiation. In contrast, the addition of GDOWN1 to ncPol II reduces transcription initiation by ~80%. When combining GDOWN1 with RPAP2 or the complex of RPAP2 with GPN1 and GPN3, transcription initiation is undetectable (Fig. 4d, e). This is consistent with GDOWN1 competing with GTFs and promoter DNA, thereby inhibiting transcription. Moreover, the inhibitory effect of GDOWN1 was less pronounced when a phosphomimetic S270E mutant was used (Fig. 4d, e), which aligns with previous reports showing that phosphorylation of S270 alleviates transcription inhibition by GDOWN1^25^.

Altogether, our data show that cfPol II bound by GDOWN1 and RPAP2 is unable to initiate transcription and that its inhibition is mainly mediated by GDOWN1.

## Discussion

In conclusion, our structure of cfPol II provides the atomic model for GDOWN1 bound to Pol II. It shows three distinct regions of GDOWN1 binding to RPB10 as well as the external and protrusion domains of Pol II. GDOWN1 binds to RPB3, RPB10, and RPB12 and helps to assemble an RPB3-containing Pol II subassembly. Binding of GDOWN1 to Pol II also sterically clashes with transcription initiation factors TFIIB and TFIIF as well as promoter DNA, and cfPol II is unable to initiate transcription in vitro. Together with previous studies, our findings establish the basis for GDOWN1-dependent transcription repression and suggest a model for how GDOWN1 may facilitate Pol II assembly and import, and may rapidly be exported upon dissociation from Pol II^2^.

During Pol II assembly, GDOWN1 binds the subcomplex comprising RPB2, RPB3, RPB10, RPB11 and RPB12^14^, and binding of GDOWN1 to cfPol II bridges between Pol II subunits RPB3, RPB10 and RPB12 as well as RPB2 and RPB3 (Fig. 2b). Based on similarity to the assembly pathway of bacterial RNA polymerase, RPB2 and RPB3 have been hypothesized to be part of separate subcomplexes that then associate to form the RPB2, RPB9, RPB3, RPB10, RPB11 and RPB12 subassembly at an early step of Pol II assembly^7^. Our data support a model in which GDOWN1 may stabilize the RPB3 subassembly before its association with the RPB2-RPB9 complex, and we therefore propose that GDOWN1 may stabilize the formation of this subcomplex and thereby function during cytoplasmic Pol II assembly.

GDOWN1 binds to Pol II before nuclear import, as evident from our isolation of cfPol II. The GDOWN1 C-terminus contains a cytoplasmic anchoring signal (CAS) that was shown to facilitate localization to the nuclear pore by interacting with RAE1 and NUP50 at the cytoplasmic and nuclear side of the pore, respectively^2^. This indicates that GDOWN1, when bound to cfPol II, can facilitate nuclear import of Pol II.

GDOWN1 is imported to the nucleus alongside Pol II^7^, and nuclear accumulation of GDOWN1 was shown to correlate with low transcriptional activity^30^ and cause global repression of transcription^2^. Our structural and functional data explain how GDOWN1 acts as a repressor of transcription by interfering with PIC assembly. Moreover, the GDOWN1 binding site on Pol II also overlaps with the binding sites of elongation, pausing, and termination factors PAF1, LEO1, RTF1, NELF-A, ELL2, and TTF2 (Supplementary Fig. 10), further suggesting that GDOWN1 can also interfere with the nuclear function of these factors. Altogether, our structural and biochemical data substantiate the role of GDOWN1 as a global repressor of transcription in the nucleus.

To render nuclear Pol II transcriptionally active, GDOWN1 needs to be released and exported to the cytoplasm, where the majority of GDOWN1 localizes^2,18^. Recent studies showed that phosphorylation of GDOWN1 at S270^25^, likely by the Mediator kinase CDK8, relieves the inhibition of transcription initiation^19,23,35^. Our biochemical data reveal that phosphorylation of S270 reduces the inhibitory effect of GDOWN1 on transcription initiation. However, in our structure, S270 resides in the unresolved part between the EBR and PBR, which precludes from understanding how phosphorylation of GDOWN1 regulates Pol II association.

GDOWN1 comprises two nuclear export sequences (NES)^18^ that facilitate the rapid nuclear export of free GDOWN1. One of the two NES resides at the C-terminal end (residues 332–340) of the helix α5 of PBR and interacts with the Pol II protrusion (Fig. 2). This NES may only become accessible after GDOWN1 release from Pol II, suggesting a tightly regulated relationship between GDOWN1 release from Pol II and its nuclear export. This regulation may ensure that GDOWN1 is exported from the nucleus only under specific conditions, which may be crucial for its cellular function.

Altogether, our structural and functional data propose a model by which GDOWN1 may facilitate Pol II assembly and import, act as a global transcription repressor in the nucleus, and may rapidly be exported upon dissociation from Pol II. Our work establishes the structural framework to study GDOWN1 function, and paves the way for future studies investigating the molecular mechanisms by which GDOWN1 controls Pol II assembly and nuclear import, and facilitates transcription repression in the nucleus.

## Methods

### Molecular cloning

The cDNAs encoding full-length human assembly and transport factors GPN1 and GPN3 were amplified from a human cDNA library using following primers gpn1_fwd: 5′-TACTTCCAATCCAATGCAATGGCGGCGTCCGCAG-3′, gpn1_rev: 5′-TTATCCACTTCCAATGTTATTATTTATTGTTTCTCTTCCAGTATTGTGCCATCG-3′, gpn3_fwd: 5′-TACTTCCAATCCAATGCAATGCCTCGGTATGCGCAGCT-3′, gpn3_rev: 5′-TTATCCACTTCCAATGTTATTATTTATTGTTTCTCTTCCAGTATTGTGCCATCG-3′. The amplified GPN1 and GPN3 cDNAs were cloned separately into the p438C vector, which encodes proteins with an N-terminal His₆-MBP tag. Further, codon-optimized cDNAs for expression in Hi5 (*Trichoplusia ni*) encoding full-length human RPB2, RPB3, RPB9, RPB10, RPB11, and RPB12, cloned in p438A (IDT), were cleaved using PmeI or SwaI as needed for insertion into the p438A or p438C vectors. This approach yielded the following constructs: p438A-RPB3-11-12-10 (no of human Pol II subunits contained a tag), p438A-RPB12-10 (no tag), and p438A-RPB2-9 (no tag). Furthermore, the construct p438C-RPB3-RPB11 was prepared, which includes an N-terminal His₆-MBP tag on RPB3. The cDNA coding for human GDOWN1 was amplified from the construct pOPINB_plus_GDOWN1_FL using the primers: GDOWN1-for: 5′-TACTTCCAATCCAATGCAATGTGTAGCCTGCCTCGTGG-3′ and GDOWN1-rev: 5′-TTATCCACTTCCAATGTTATTAGAATTCATCACTGCTCCAATCATCATCCTC-3′. The amplified cDNA was cloned into the p438C vector, resulting in a construct coding for N-terminal His₆-MBP tagged human GDOWN1. To generate the phosphomimetic mutant of GDOWN1 (S270E), the wild-type GDOWN1 was first cloned into the pGEX6P1 vector using the primers: GDOWN1-for: 5′-CGAATTGGATCCATGTGTAGCCTGCCTCGTGG-3′ (BamHI) and GDOWN1-rev: 5′-CATTTATCTCGAGTAGAATTCATCACTGCTCCAATC- 3′(XhoI). This construct expresses N-terminal GST-tagged GDOWN1. Site -directed mutagenesis was then performed to introduce the S270E mutation using the primers: for: 5′- CATTGTCAGAAAAGCGGTGAACCGATTAGCAGTGAAGAAC-3′ and rev: 5′-GTTCTTCACTGCTAATCGGTTCACCGCTTTTCTGACAATG-3′.

### Purification of Pol II from the cytoplasm

All protein purification steps were performed at 4˚C unless otherwise specified. Pol II was purified from the cytoplasmic fraction of HEK293 cells stably expressing N-terminally His_10_-Twin-Strep-tagged human Pol II subunit RPB1, generated via CRISPR/Cas9 genome editing^31^. Cells were grown in a 15 L fermenter (Applikon BioBundle, 15 L capacity, ez2-control) to a density of 3–6 × 10⁶ cells/mL with viability between 86 and 93%.

The cytoplasmic fraction was prepared according to the method of Dignam et al.^36^. After harvesting, cells were washed twice with 1x PBS buffer and pelleted by centrifugation at 836 × *g* for 10 min. The cell pellet was resuspended in hypotonic 1× MC buffer (10 mM HEPES-KOH, pH 7.6; 10 mM KOAc; 0.5 mM Mg(OAc)₂; 0.5 mM DTT), supplemented with EDTA-free protease inhibitors (Roche; 2 tablets per 50 mL buffer), and incubated on ice for 5 min. Swollen cells were lysed using a 100 mL Dounce homogenizer (Kimble Chase, Sigma-Aldrich) with 18 strokes, followed by centrifugation at 18,000 × *g* for 5 min. The supernatant (cytosolic extract) was flash-frozen and stored at −80 °C.

Prior to purification, the thawed cytoplasmic fraction was cleared by centrifugation at 26,074 × *g* for 30 min (rotor Fiberlite F14 6x250y, Thermo Fisher Scientific). To remove nucleic acid contaminants, the supernatant was treated with 5% polyethylenimine (PEI, pH 7.9) to a final concentration of 0.04%. After 10 min of stirring at 4 °C, the resulting white precipitate was pelleted by centrifugation at 18,668 × *g* for 20 min. The pellet was washed with A0 buffer (50 mM Tris-HCl, pH 7.9; 5 mM MgCl₂; 2 mM DTT; 10% glycerol) using 5× Dounce homogenization followed by centrifugation at 18,668 × *g* for 20 min. The washed pellet was resuspended in buffer A100 (50 mM Tris-HCl, pH 7.9; 1.5 mM MgCl₂; 2 mM DTT; 10% glycerol; 100 mM ammonium sulfate) by stirring for 60 min at 4 °C.

Ammonium sulfate precipitation was performed by adding finely ground ammonium sulfate to achieve 50% saturation. The mixture was gently stirred at 4 °C overnight. The pellet was obtained by centrifugation at 20,217 × *g*. Further, the pellet was resuspended in A0 buffer (by stirring for 60 min at 4 °C) and cleared by centrifugation at 74,766 × *g* (rotor A27 6 × 50, Thermo Fisher Scientific) for 60 min. The supernatant was filtered through a 45 μm Filtropur S filter (Sarstedt) and loaded onto a 20 mL self-packed Strep-TactinXT 4Flow column (IBA Life Sciences), which had been equilibrated in buffer A100. The column was washed with 10 column volumes of buffer A100 and eluted with 50 mM biotin (IBA Life Sciences) in buffer A100. All buffers were supplemented with protease inhibitors: 1 mM PMSF, 1 mM benzamidine, 60 μM leupeptin, and 200 μM pepstatin.

The eluted fractions were analyzed by SDS-PAGE using a 4–12% Bis-Tris NuPAGE gel in 1× MES running buffer (Invitrogen). Fractions containing cfPol II associated with assembly factors were pooled and concentrated using Amicon Ultra centrifugal filters (100 kDa molecular weight cutoff, MilliporeSigma). From approximately 32 × 10^9^ cells, about 150 μg of cfPol II were purified. Samples for cryo-EM analysis, with or without spike-in of recombinantly expressed and isolated assembly factors, were further purified by size-exclusion chromatography on a Superose 6 Increase column (3.2 × 300 mm, GE Healthcare). Fractions corresponding to monomeric Pol II complexed with assembly factors were pooled, concentrated, and used for further experiments.

### Purification of the RPAP2-GPN1-GPN3 complex

To facilitate expression of the individual N-terminal His₆-MBP-tagged proteins (RPAP2^27^, GPN1, and GPN3), bacmids were generated using DH10αEMBacY cells. SF9 cells (*Spodoptera frugiperda*; Thermo Fisher Scientific) were transfected with the corresponding bacmids to produce V0 virus, which was then used to generate V1 virus in SF9 cells. Large-scale co-expression of RPAP2-GPN1-GPN3 was carried out in Hi5 cells (*Trichoplusia ni*.; Expression Systems) via co-transfection with V1 viruses encoding each protein subunit. The insect cells expressing the recombinant proteins were harvested at 85–90% viability to minimize degradation.

Frozen cell pellets were thawed at room temperature, resuspended in the lysis buffer A250 (50 mM Tris-HCl, pH 7.9; 250 mM ammonium sulfate; 2.5 mM MgCl_2_; 10% glycerol; 0.1 mM EDTA, pH 8.0; 2 mM DTT; and protease inhibitors: 1 mM PMSF, 1 mM benzamidine, 60 μM leupeptin, and 200 μM pepstatin), and lysed by sonication.

After centrifugation at 74,766 × *g*, for 60 min (rotor A27 6 × 50, Thermo Fisher Scientific), the supernatant was filtered sequentially through 0.8 μm and 0.45μm Filtropur S filter (Sarstedt) to remove aggregates. The clarified lysate was then incubated with 10 ml amylose resin (NEB) in batch, pre-equilibrated with lysis buffer A250. The complex was eluted by on-resin cleavage with TEV protease overnight at 4 °C. The resulting assembled heterotrimeric complex was further purified on a Sepharose 200 10/300 column (Cytiva), equilibrated in buffer A150 (HEPES-NaOH pH 7.5, 150 mM NaCl, 2 mM DTT, 5% glycerol).

### Purification of full-length human RPAP2

Full-length 6xHis-MBP-RPAP2 was expressed in insect cells using the p438C-RPAP2 construct^27^. Production of bacmid, V_0_ and V_1_ baculoviruses, and protein expression were performed as written above (Methods/Purification of RPAP2, GPN1, and GPN3). Hi5 cells expressing full-length RPAP2 were harvested by centrifugation, flash frozen in liquid nitrogen, and stored at −70 °C. Frozen pellets of Hi5 cells were thawed at room temperature, resuspended in lysis buffer (50 mM Tris-HCl, pH 8; 500 mM NaCl; 30 mM imidazole, pH 8; 10% glycerol; 5 mM β-mercaptoethanol (BME); 10 μM ZnCl_2_; 1 mM PMSF; 1 mM benzamidine; 60 μM leupeptin; and 200 μM pepstatin) at a ratio of 20 mL buffer per gram of cells, and lysed by sonication. The lysate was cleared by centrifugation at 4 °C and 47,850 × *g* for 1 h, filtered through 0.45 μm syringe filters (Merck Millipore), and incubated for 1 h at 4 °C with Ni-NTA beads (Qiagen) pre-equilibrated in lysis buffer. The beads were washed with lysis buffer and 10% Buffer B (50 mM Tris-HCl, pH 8; 200 mM NaCl; 300 mM imidazole, pH 8; 10% glycerol; 5 mM β-mercaptoethanol; 10 μM ZnCl_2_). The 6xHis-MBP-tagged RPAP2 was eluted with 50% and 100% Buffer B1. The wash and elutions were pooled and incubated for 1 h with amylose beads (NEB) in batch, pre-equilibrated in lysis buffer. The tag-free RPAP2 was eluted by on-resin cleavage with TEV protease overnight at 4 °C. The TEV protease and the tag were removed by incubating the eluate with Ni-NTA beads. The sample was further purified using a 5 ml pre-packed SP-Sepharose HP column. Peak fractions containing the purified protein were pooled, concentrated using a 50,000 MWCO Amicon Ultra Centrifugal Filter (Merck Millipore), aliquoted, snap-frozen in liquid nitrogen, and stored at −70 °C.

### Pull-down analysis of interactions between human GDOWN1 and human RPB2-RPB3 subcomplexes

To facilitate the expression of different RPB2 and RPB3 subcomplexes with and without 6xHisMBP-tagged GDOWN1, the preparation of bacmids and the production of V1 were performed as described above (Methods/Purification of RPAP2, GPN1, and GPN3). Large-scale co-expression (600 mL cultures in 3 L flasks) of the complexes was carried out in Hi5 cells (Expression Systems) via co-transfection with V1 viruses encoding the corresponding subcomplexes. Smaller-scale co-expression (100–200 mL of culture in 1 L flasks) was tested, but resulted in poor expression. Additionally, expression in SF21 cells was attempted, but some subunits of the subcomplexes were not expressed in this strain. Insect cells expressing the recombinant proteins were harvested at 85–90% viability to minimize degradation. Frozen cell pellets were thawed at room temperature, resuspended in lysis buffer A100 (50 mM HEPES, pH 7.6; 100 mM ammonium sulfate; 5 mM MgCl_2_; 5 mM ATP; 0.5 mM CaCl_2_; 10 μM ZnCl_2_; 10% glycerol; 2 mM DTT; and protease inhibitors: 1 mM PMSF, 1 mM benzamidine, 60 μM leupeptin, and 200 μM pepstatin), and lysed by sonication. The lysate was then incubated with DNase I for 15 min at room temperature.

After centrifugation at 74,766 × *g* for 60 min (rotor A27 6 × 50, Thermo Fisher Scientific), the supernatant was filtered sequentially through 0.8 μm and 0.45 μm Filtropur S filters (Sarstedt) to remove aggregates. The clarified lysate was then incubated for 3 h with 10 ml of amylose resin (NEB), pre-equilibrated with lysis buffer A100, using end-over-end incubation. After extensive washing of the beads with lysis buffer, the bound proteins were analyzed by denaturing gradient gel electrophoresis (4–12% Bis-Tris NuPAGE, Life Technologies).

### Purification of full-length human GDOWN1

Human N-terminal His₆ tagged GDOWN1 was expressed from plasmid pOPINB_plus_GDOWN1_FL^32^ in *E. coli* BL21-RIL cells by induction at OD_600_ = 1.2 with 1 mM IPTG for 18 h at 18˚C. Frozen cell pellets were thawed at room temperature, resuspended in the lysis buffer A400 (50 mM HEPES-NaOH, pH 7.5; 400 mM NaCl; 30 mM imidazole; 10% glycerol; 2 mM BME; and protease inhibitors: 1 mM PMSF, 1 mM benzamidine, 60 μM leupeptin, and 200 μM pepstatin), and lysed by sonication. The lysate was cleared by centrifugation at 74,766 × *g* (rotor A27 6 × 50, Thermo Fisher Scientific) for 60 min and filtered with 0.45 μm Filtropur S filter (Sarstedt) to remove aggregates. The clarified supernatant was loaded onto a 5 ml His Trap HP column (Cytiva), pre-equilibrated with lysis buffer A400. His_6_-GDOWN1 was eluted with linear imidazole gradient from 30 mM to 400 mM in buffer A200 (25 mM HEPES-NaOH, pH 7.5; 200 mM NaCl; 10% glycerol; 2 mM BME). Fractions containing His_6_-GDOWN1 were pooled, and the His_6_ tag was removed by incubation with TEV protease overnight at 4˚C during dialysis against buffer A100 (25 mM HEPES-NaOH, pH 7.5; 100 mM NaCl; 10% glycerol; 2 mM BME). The tag-free GDOWN1 was further purified by reverse affinity chromatography: the dialyzed sample was incubated with Ni-NTA resin (Qiagen) in batch to remove the cleaved His_6_-tag and residual His_6_-tagged proteins. The flow-through, enriched in tag-free GDOWN1, was then applied to a 5 ml Q -Sepharose HP column (Cytiva), equilibrated in buffer A100. Proteins were eluted using a linear NaCl gradient from 0.1 to 1 M. Fractions containing pure, contaminant-free GDOWN1 were pooled, concentrated, and flash-frozen for storage at −80 °C.

### Purification of full-length human phosphomimetic GDOWN1

Human N-terminal GST-tagged phosphomimetic GDOWN1 was expressed from the plasmid pGEX6P1-GDOWN1-S270E in *E. coli* BL21-RIL cells. Expression was induced at an OD_600_ = 0.8 with 0.1 mM IPTG for 18 h at 18 °C. A frozen cell pellet from 1 L culture was thawed at room temperature, resuspended in lysis buffer A400 (50 mM HEPES-NaOH, pH 7.5; 400 mM NaCl; 5 mM MgCl_2_;5 mM ATP; 0.5 mM CaCl_2_;10% glycerol; 2 mM BME; and protease inhibitors: 1 mM PMSF, 1 mM benzamidine, 60 μM leupeptin, and 200 μM pepstatin), and lysed by sonication. After sonication, the cell lysate was incubated with DNase I (2 μg/ml) for 15 min at room temperature. The lysate was clarified by centrifugation at 74,766 × *g* (rotor A27 6 × 50, Thermo Fisher Scientific) for 60 min and filtered with 0.45 μm Filtropur S filter (Sarstedt) to remove aggregates. The clarified supernatant was loaded onto 2 × 5 ml Glutathione (GSH) Sepharose HP columns (Cytiva), pre-equilibrated with lysis buffer A400. Bound GST-GDOWN1-S270E was washed with A400 and then with A1M (25 mM HEPES-NaOH, pH 7.5; 1 M NaCl; 10% glycerol; 2 mM BME) and further equilibrated with buffer A150 (25 mM HEPES-NaOH, pH 7.5; 150 mM NaCl; 10% glycerol; 2 mM BME). The protein was eluted with 20 mM reduced glutathione in buffer A150. Fractions containing GST-GDOWN1 were pooled and applied to a 5 ml Q -Sepharose HP column (Cytiva), equilibrated in buffer A100 (25 mM HEPES-NaOH, pH 7.5; 100 mM NaCl; 10% glycerol; 2 mM BME). The GST tagged GDOWN1 S270E was eluted using a linear NaCl gradient from 0.1 to 1 M.

The GST tag was removed by incubation with 3C protease overnight at 4 °C during dialysis against buffer A100 (25 mM HEPES-NaOH, pH 7.5; 100 mM NaCl; 10% glycerol; 2 mM BME). The tag-free GDOWN1 was further purified by reverse affinity chromatography: the sample was incubated with GSH resin (Cytiva). Afterwards, the 3C protease was removed by incubating the protein sample with NI-NTA beads (Qiagen) in batch to remove the His-tagged 3C protease. The flow-through, enriched in tag-free GDOWN1 S270E mutant protein, was further purified using a Q -Sepharose HP column (Cytiva), equilibrated in buffer A100. Proteins were eluted using a linear NaCl gradient from 0.1 to 1 M. Fractions containing pure, contaminant, and tag -free GDOWN1 S270E were pooled, concentrated, and flash-frozen for storage at −80 °C.

### Purification of human general transcription factors

Human GTFs, including TBP, TFIIA, TFIIB, TFIIE, TFIIF, and TFIIH, were expressed and purified as previously described^37,38^. Specifically, TFIIA, TFIIH, and TBP were independently expressed in insect cells, while TFIIB, TFIIE, and TFIIF were expressed in LOBSTR-BL21(DE3)-RIL E. coli and BL21-Codon Plus(DE3)-RIL E. coli cells, respectively. Upon expression, frozen cell pellets were thawed in a water bath at 25 °C. *E. coli* cells were lysed by sonication, and insect cells were supplemented with DNase I before lysis using an EmulsiFlex-C5 cell disruptor (Avestin) (3 passages, 12,000 psi). Cell lysates were clarified by centrifugation (79,000 × *g*; 60 min), and the soluble fraction was filtered through 0.45-μm syringe filters (Merck Millipore). All purification procedures were conducted at 4 °C. Purification buffers were filtered and degassed.

The initial purification of TFIIA, TFIIB, TBP, TFIIE, and TFIIF involved GE HisTrap HP (5 mL) affinity chromatography, followed by ion exchange and size exclusion chromatography steps.

### Cryo-EM sample preparation

Approximately 150 pmol of cfPol II were incubated with a 2-fold molar excess of recombinantly expressed, tag-free assembly factors (RPAP2, GPN1, GPN3, and GDOWN1) for 5 min at 4 °C. The resulting complex was purified by size-exclusion chromatography on a Superose 6 Increase column 3.2/300 (GE Healthcare), pre-equilibrated in buffer A150 (20 mM HEPES-NaOH, pH 7.5, 150 mM NaCl, 0.5 mM TCEP, 5% glycerol). Peak fractions enriched in cfPol II containing the spiked-in assembly factors were pooled from four independent runs and concentrated to 1.56 μM. A 100 μL aliquot of the sample was further crosslinked with 0.75 mM BS3 (bis(sulfosuccimidyl)suberate; Thermo Fisher Scientific) and incubated on ice for 30 min. The reaction was quenched by adding pH-adjusted Tris to 100 mM final concentration, followed by an additional 1-h incubation at 4 °C. The sample was then buffer-exchanged into A150 (without glycerol) by dialysing for 5 h at 4 °C using a 10,000 MWCO Slide-A-Lyzer MINI Dialysis Unit (Thermo Fisher Scientific). The resulting crosslinked cfPol II complex (Pol II-RPAP2-GPN1-GPN3-GDOWN1) was used for cryo-EM grid preparation. A volume of 3.5 μL of the diluted sample (~600 nM) was applied to one side of freshly glow-discharged R2/2 UltrAuFoil 200 grids (Quantifoil). Grids were blotted for 5 s at a blot force of 5, at 4 °C and 98% humidity, and rapidly vitrified by plunging into liquid ethane using a Vitrobot Mark IV (Thermo Fisher Scientific).

### Chemical crosslinking mass spectrometry

For dataset 1, 75 pmol of the purified endogenous cfPol II complexes, after size exclusion chromatography (without spike-in of the assembly factors), were crosslinked with either 0.7 mM or 1.4 mM BS3 (Thermo Fisher) for 30 min at 24 °C in 50 µl of buffer composed of 20 mM HEPES-NaOH, pH 7.5, 150 mM NaCl, 1.5 mM MgCl_2_, 2 mM DTT. The crosslinking reactions were quenched with 55 mM Tris-HCl, pH 7.5, for 15 min at 24 °C. For dataset 2, endogenous cfPol II complexes were spiked-in with recombinant human assembly factors (GDOWN1, RPAP2, GPN1, and GPN3), and the excess of the recombinant proteins was removed by size exclusion chromatography using a Superose 6 Increase column (Cytiva). 100 pmol of these cfPol II complexes were crosslinked with 2 mM BS3 (Thermo Fisher) for 30 min at 24 °C in 50 µl of the buffer composed of 20 mM HEPES-NaOH, pH 7.5, 150 mM NaCl, 1.5 mM MgCl_2_, 2 mM DTT. Both samples were subsequently denatured with 4 M urea, reduced with 5 mM DTT, and alkylated with 17 mM iodoacetamide.

The samples were then diluted with 50 mM ammonium bicarbonate to reduce urea concentration to 1 M and digested with trypsin (Promega, sequencing grade) in a 1:20 enzyme-to-protein ratio (w/w) at 37 °C overnight. Peptides were reverse-phase extracted using SepPak Vac tC18 1cc/50 mg (Waters), eluted with 50% acetonitrile (ACN)/0.1% trifluoroacetic acid (TFA), and dried in a vacuum concentrator (Eppendorf). Obtained peptides were dissolved in 40 µl of 2% ACN / 20 mM ammonium hydroxide and separated by reverse phase HPLC at basic pH using an xBridge C18 3.5 µm 1 × 150 mm column (Waters) at a flow rate of 60 µl/min at 24 °C. A gradient of buffers A (20 mM ammonium hydroxide, pH 10) and B (80% ACN / 20 mM ammonium hydroxide, pH 10) was used as mobile phase. Peptides were bound to a column pre-equilibrated with 5 % buffer B and eluted over 64 min using a multistep gradient of 5-45%B. Fractions of 60 µl were collected. Peptides eluted between 10 and 56 min were pooled with a step of 15 min, vacuum dried, and dissolved in 5% ACN / 0.1% TFA for a subsequent uHPLC-ESI-MS/MS analysis. The fractions were injected into a Dionex UltiMate 3000 uHPLC system coupled to an Orbitrap Exploris 480 mass spectrometer (both Thermo Fischer) and measured with a 75 min methods 4 or 3 times (the datasets 1 and 2, respectively). uHPLC was equipped with a C18 PepMap or PepMap Neo Trap cartridge (0.3 × 5 mm, 5 μm, Thermo Scientific) and a custom 30 cm C18 main column (75 µm inner diameter packed with ReproSil-Pur 120 C18-AQ beads, 3 µm pore size, Dr. Maisch GmbH). Mobile phase was formed using buffers A (0.1% formic acid) and B (80% ACN/0.08% formic acid). Peptides were separated by applying a linear multistep gradient of 10-52%B. MS settings were as follows: MS1 resolution, 120,000; MS1 scan range, 350–1550 *m*/*z*; MS1 normalized AGC target, 300%; MS1 maximum injection time, 25 ms; cycle time (Top Speed), 3 s; intensity threshold, 1E4; MS2 resolution, 30000; isolation window, 1.6 Th; normalized collision energy, 28%; MS2 AGC target, 75%; MS2 maximum injection time, 128 ms. Only precursors with a charge state of 3–8 were selected for MS2 using a dynamic exclusion of 20 s.

Protein composition of the samples was determined in a MASCOT v.2.3.02 search against UniProt human reference proteome (release 2022-10-12) supplemented with common contaminants observed in proteomics experiments and the sequence of the tagged version of RPB1. Tryptic peptides were considered with a maximum of 3 missed cleavages. Methionine oxidation, cysteine carbamidomethylation, and DSS-monolinks of lysine and protein N-termini were set as variable modifications. MS1 and MS2 tolerances were 10 ppm and 20 mDa, respectively. Proteins identified by a minimum of 2 peptides (spectral and protein level false discovery rate (FDR) of 0.03 and 0.4%, respectively) were considered. Using a spectrum counting approach (the number of observed peptide-spectrum matches normalized to the protein size), restricted databases were created to encompass either 240 most abundant proteins (dataset 1) or all 387 identified proteins (dataset 2). The former database was used for a protein-protein crosslink search of Thermo raw files using pLink 2.3.11 (dataset 1), and the latter for a pLink 3.0.17 search (both datasets 1 and 2) (https://pfind.ict.ac.cn/se/plink/)^39^. The pLink results were filtered at an FDR of either 1 or 5% at the spectral level (pLink 2.3.11) or peptide-pair level (pLink 3.0.17) and presented in Supplementary Tables 1 and 2, respectively. For model building, a maximum distance of 30 Å between the Cα atoms of the crosslinked lysines was allowed.

### Western blot analysis of cell fractionation

Approximately 15 µg of protein samples from cytoplasmic or nuclear fractionations, obtained after cell fractionation according to Dignam^36^, were loaded into wells and separated with denaturing gradient gel electrophoresis (4–20% Bis-Tris Nupage, Life Technologies). The gels were transferred onto a nitrocellulose membrane (GE Healthcare Life Sciences) for Western blotting, which was carried out using standard protocols. To visualize human RPB1 (Supplementary Fig. 1b), which is N-terminally tagged with Twin-Strep and His-tags, an antibody against Twin-Strep (ab76949, Fischer Scientific) was used.

### Cryo-EM data collection and processing

Cryo-EM data were collected on a 300 kV Titan Krios transmission electron microscope (Thermo Fisher Scientific) equipped with a K3 direct electron detector (Gatan) and a Quantum LS energy filter. Data were acquired in electron counting mode at a nominal magnification of ×81,000, corresponding to a physical pixel size of 1.05 Å/pixel. A 20 eV energy filter slit was used in EFTEM mode. Automated data collection was performed using SerialEM^40^. Exposure time was 2.316 s, with a dose rate of 19.0 e⁻/pixel/s (17.23 e⁻/Å²/s), resulting in a total dose of 39.91 e⁻/Å². The defocus range was set between −0.5 and −2.0 μm. A total of ~11,000 micrographs were collected.

All movie stacks were processed on-the-fly using Warp^41^ for motion correction, dose weighting, and contrast transfer function (CTF) estimation. Micrographs with poor CTF estimates were excluded from further analysis. Approximately 4.1 million particles were extracted using a box size of 280 pixels (binned 2×, resulting in a pixel size of 2.1 Å/pixel) in RELION 5.0^42,43^.

Particles were subjected to iterative rounds of 2D and 3D classification in CryoSPARC^44^. Classes showing well-defined features of Pol II and assembly factors, including the Pol II stalk, the RPAP2 N-terminal domain, and the C-terminal region of GDOWN1, were selected for further processing. These particles (~2.4 million) were re-extracted in their original, unbinned form (pixel size: 1.05 Å/pixel) and further processed in RELION 5.0. Iterative rounds of CTF refinement, 3D refinement, and Bayesian polishing were performed, resulting in a final 3D reconstruction of the Pol II core at a resolution of 2.3 Å.

To improve the resolution for the density corresponding to GDOWN1, a soft mask encompassing protrusion, lobe and wall of RPB2, GDOWN1 C- and N-termini, RBP10, RPB12 and part of RPB3 was applied to focus 3D classification on 2.4 million particles without image alignment using blush function and with regularization parameter *T* = 4 (Supplementary Fig. 2). The 3D refinement of the best class containing 265,000 particles (11%) showing C-terminal and partially N-terminal part of GDOWN1 was performed. This map was further used for new soft mask preparation encompassing N-terminal GDOWN1, RPB10, RPB12, the C-terminal part of RPB3, as well as parts of the N-termini, lobe, and wall of RPB2, respectively. New focus 3D classification without image alignment using the blush function and with regularization parameter *T* = 4, with this soft mask was performed on 2.4 million particles. The best class, containing 472,100 particles, was further 3D refined, obtaining a reconstruction of the map with 2.6 Å resolution of the core Pol II. Another round of focus 3D classification was performed to get a better map for the N-termini of GDOWN1. Here, two classes, soft mask on the region of N-termini of GDOWN1 were used. Further 3D refinement of the best class (188,000 particles), a 2.7 Å reconstruction was obtained. To improve the resolution of the C-terminus of GDOWN1, a soft mask for the region corresponding to RPB12, of protrusion, lobe, and wall of RPB2 was used for focus classification with two classes, blush and *T* = 4. The best class (47, 500 particles) was 3D focus refined, resulting in 2.9 Å resolution of the core Pol II (Map A). Resolution ranges for the map regions corresponding to the GDOWN1 N-terminus (amino acids 19–73), the central part (amino acids 224–255), and the C-terminus (amino acids 290–334) were 3.3–4.1, 3.1–3.4, and 2.9–4.7 Å, respectively.

The resolution of the N-terminal part of RPAP2 in Map A was limited to 3.8–5.3 Å. Consequently, we performed additional processing focused on RPAP2, resulting in Map B (Supplementary Fig. 3). We conducted focus 3D classification using a mask encompassing the N-terminal part of RPAP2 and the jaw domain of RPB1 on 2.4 million particles without image alignment, applying blush regularization and two classes. The class exhibiting well-defined N-terminal RPAP2 density (584,000 particles) was subjected to global 3D refinement. A second round of 3D classification under identical masking and parameters using three classes yielded a class (214,000 particles) that was refined to Map B. This map achieved a resolution range of 3.1–4.2 Å around the N-terminal RPAP2 and 2.6 Å for the core Pol II.

### Model building

For model building, Pol II from the Pol II dimer model (PDB: 7OZN^45^) was rigid-body docked into Map A in Chimera^46^. The starting models for full-length GDOWN1 and RPAP2 (residues 1–401) bound to Pol II (RPB1 with residues 1–1546; all other subunits of Pol II are full length) were generated by AlphaFold 3^33^. To model RPAP2, the initial model was docked into Map B, which has better density for RPAP2, and manually rebuilt in Coot^47^and real-space refined with Phenix^48^. The refined RPAP2 model and the starting models of Pol II and GDOWN1 were fitted as rigid bodies into Map A. The Pol II-RPAP2-GDOWN1 model was then manually adjusted in Coot^47^ against Map A, followed by consecutive rounds of real-space refinement in Phenix^48^ and rebuilding with Coot^47^. The final Pol II-RPAP2-GDOWN1 model showed good stereochemistry as validated by Molprobity^49^.

### In vitro transcription initiation assay

GTFs and human nfPol II were purified as previously described^31,32,37,38,50,51^. In vitro transcription initiation assays were performed as previously described^34,52^. In brief, promoter templates were generated by large-scale PCR, followed by ion-exchange chromatography and isopropanol precipitation. For each reaction, 1.1 pmol Pol II, 5.8 pmol TFIIF, 1.2 pmol DNA, 3.8 pmol TBP, 7.7 pmol TFIIA, 3.8 pmol TFIIB, 1.7 pmol TFIIE, and 1.7 pmol TFIIH were used. PIC was assembled in a reaction buffer containing 20 mM HEPES-KOH, pH 7.5, 109 mM KCl, 3% glycerol, 6 mM MgCl_2_, 0.5 mM DTT at 30˚C for 60 min. Transcription was initiated with the addition of 0.5 mM ATP, 0.5 mM CTP, and 0.5 mM GTP and allowed to proceed for 10 min at 30 °C. RNAs were purified by proteinase K digestion and isopropanol precipitation and analyzed on a urea-gel (7 M urea, 1x TBE, 20% acrylamide:bis-acrylamide 19:1). The signals of RNA transcripts were quantified with ImageJ 1.53, subtracted against the background, and normalized to the signal of the control experiment. Experiments were performed in at least three technical replicates.

To analyze the effect of different assembly factors on transcription initiation, 1.1 pmol nfPol II was first pre-incubated at room temperature for 10 min with GDOWN1, RPAP2, and GPN1/3 (each 23 pmol), depending on the assay conditions. Then, 5.8 pmol TFIIF was added, and the mixture was further incubated at room temperature for 10 min. Subsequent PIC assembly, transcription initiation, and RNA analysis were performed as described above.

### Reporting summary

Further information on research design is available in the Nature Portfolio Reporting Summary linked to this article.

## Supplementary information

Supplementary Information

Description of Additional Supplementary Files

Supplementary Data 1

Supplementary Data 2

Reporting Summary

Transparent Peer Review file

## Source data

Source data

## Data Availability

The cryo-EM reconstructions and final model have been deposited in the Electron Microscopy Data Bank (EMDB) under accession codes for Map A: EMD-56542 and for Map B: EMD-56541, and in the Protein Data Bank (PDB) under accession code 28JC. The starting model for Pol II was derived from PDB accession code 7OZN. The XL mass spectrometry data have been deposited to the ProteomeXchange Consortium via MassIVE partner with the dataset identifier PXD81304. Source data are provided with this paper.
