# Supplementary material for: Structure of human cytoplasmic Pol II complex explains global transcription repression by GDOWN1
Source: Nat Commun. 2026 Aug 5;17:7854. doi: 10.1038/s41467-026-76334-5 (PMC13443923; doi:10.1038/s41467-026-76334-5)
Supplement: Supplementary file 1 [file 41467_2026_76334_MOESM1_ESM.pdf]

Structure of human cytoplasmic Pol II complex explains global transcription repression by GDOWN1

Jana Schmitzová[1], Yumeng Zhan[1], Srinivasan Rengachari[1,4], Frauke Grabbe[1], Olexandr Dybkov[2], Henning Urlaub[2,3], Michael Lidschreiber[1], Christian Dienemann[1,*], Patrick Cramer[1,*]

[1] Department of Molecular Biology, Max Planck Institute for Multidisciplinary Sciences, Göttingen, Germany.
[2] Research Group Bioanalytical Mass Spectrometry, Max Planck Institute for Multidisciplinary Sciences, Göttingen, Germany.
[3] Bioanalytics, Department of Clinical Chemistry, University Medical Center, Göttingen, Germany.
[4] Current address: Section for Structural and Synthetic Biology, Department of Infectious Disease, Imperial College London, London, United Kingdom.

[*] Correspondence: christian.dienemann@mpinat.mpg.de and patrick.cramer@mpinat.mpg.de

Supplementary Figure 1-10

Supplementary Tables 1-2

Supplementary Figure 1

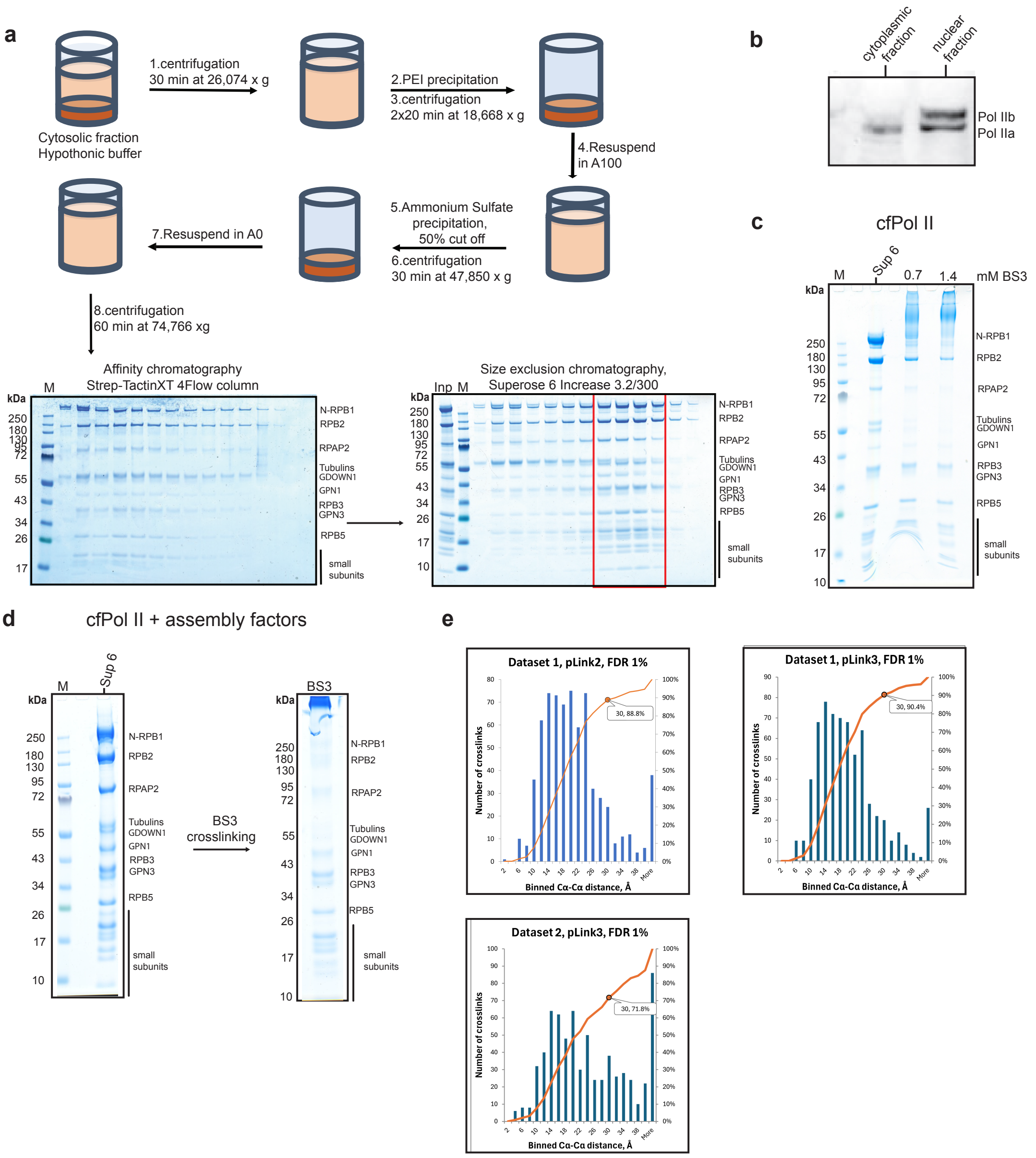

Supplementary Figure 1: Isolation of human cytoplasmic RNA polymerase II for cryo-EM analysis and crosslinking mass spectrometry

(a) Schematic of cytoplasmic human Pol II purification. Cytosolic fractions from HEK293 cells (clone C1N1 with N-terminal His10-StrepTwin tag on RPB1) were prepared following Dignam *et al.*[1]. The quality of purified cfPol II was analysed by SDS-PAGE in MES buffer to resolve all small subunits. Gels were Coomassie blue-stained. After affinity chromatography, Pol II-containing fractions were pooled, purified by size-exclusion chromatography, concentrated, and flash-frozen.

(b) Western blot analysis of cell fractionation into cytoplasmic and nuclear fractions according to Dignam[1]. For the western blot analysis, a Twin Strep antibody was used (ab76949, Fischer Scientific).
(c) SDS-PAGE analysis of endogenous cytoplasmic fraction of human Pol II after Size exclusion chromatography (Superose 6 Increase column, 3.2/300 (GE Healthcare)) and the sample after crosslinking with BS3 used for crosslinking mass spectrometry. Sup6: Concentrated pooled fraction of endogenous human cfPol II after size exclusion chromatography. BS3: BS3 crosslinked human cfPol II. Molecular weight markers (left); Pol II subunits and recombinant assembly/transport factors labelled (right).
(d) *Left:* SDS-PAGE analysis of cytoplasmic human Pol II spiked in with assembly/transport factors and purified by size-exclusion chromatography. *Right*: Sample after crosslinking with BS3. Sup6: Concentrated pooled fraction of spiked in human cfPol II after Size exclusion chromatography (column used as in Supplementary Figure 1c). BS3: BS3 crosslinked spiked in human cfPol II. Molecular weight markers (left); Pol II subunits and recombinant assembly/transport factors labelled (right).
(e) Distance distribution of the identified BS3-crosslinks mapped on the cfPol II model. The Euclidean Cα-Cα distances between the crosslinked residues were calculated in PyMOL[2]. The maximum expected threshold for BS3-crosslinks (30 Å) is indicated on the yellow line representing cumulative percentage of crosslinks. Source data are provided as a Source Data file.

Supplementary Figure 2

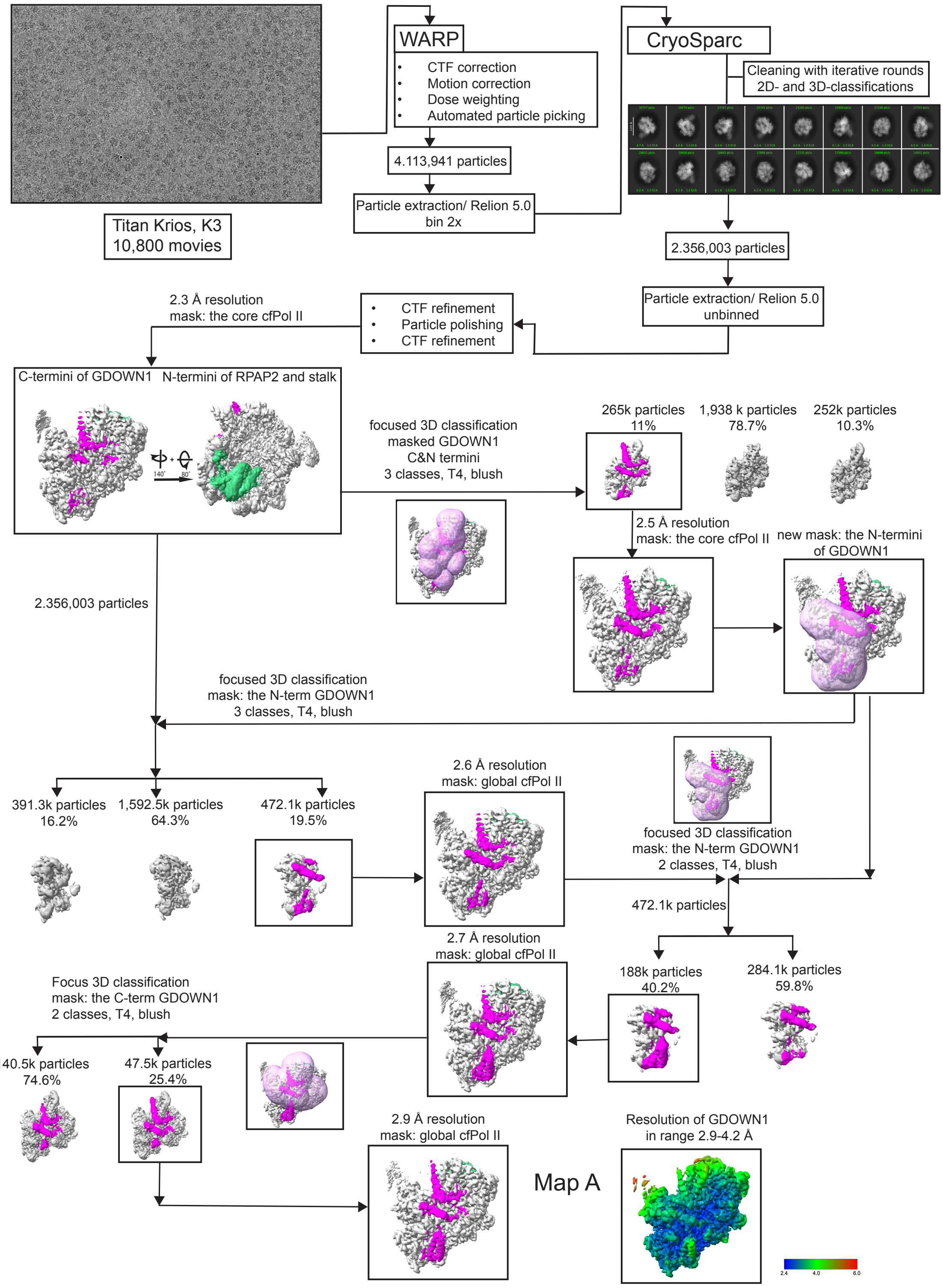

Supplementary Figure 2: Cryo-EM analysis of cytoplasmic human Pol II complexed with assembly factors RPAP2, GPN1, GPN3, and GDOWN1 (Map A; GDOWN1-focused).

The cryo-EM processing workflow begins with a representative micrograph visualized in Warp[3]. The processing tree outlines 3D classification steps and focused refinements that improved density for GDOWN1 domains. Particle classes exhibiting optimal GDOWN1 density (highlighted in pink and boxed) were selected for 3D refinement. Resulting refined maps are displayed adjacent to their corresponding 3D classification classes. Distinct extra densities are highlighted as follows: GDOWN1 at RPB2 protrusion, and at RPB10/RPB3 (magenta) and RPAP2 at RPB5 (green). Mask regions for GDOWN1-focused 3D classification appear as rosy-brown transparent surfaces. Local resolution of GDOWN1 was calculated in RELION 5.0[4,5] and visualized in ChimeraX[6].

Supplementary Figure 3

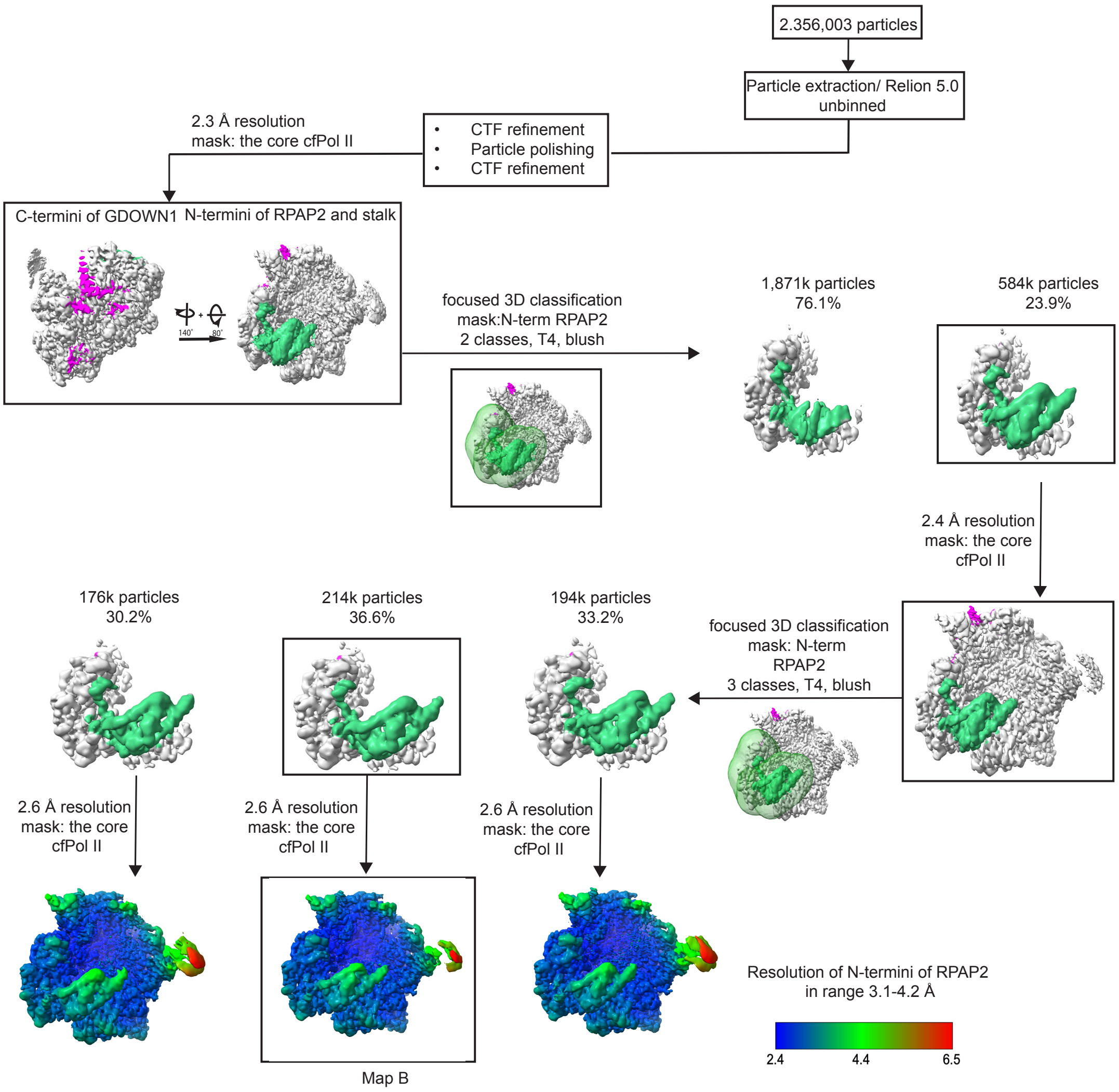

Supplementary Figure 3: Cryo-EM analysis of cytoplasmic human Pol II complexed with assembly factors RPAP2, GPN1, GPN3, and GDOWN1 (Map B; RPAP2-focused).

The cryo-EM processing workflow outlines 3D classification steps and focused refinements that improved density for the N-termini of RPAP2. Particle classes exhibiting optimal RPAP2 density (highlighted in green and boxed) were selected for 3D refinement. Resulting refined maps are displayed adjacent to their corresponding 3D classification classes. Mask regions for RPAP2-focused 3D classifications appear as light green transparent surfaces.
Local resolution maps for RPAP2 were calculated in RELION 5.0[4,5] and visualized in ChimeraX[6].

Supplementary Figure 4

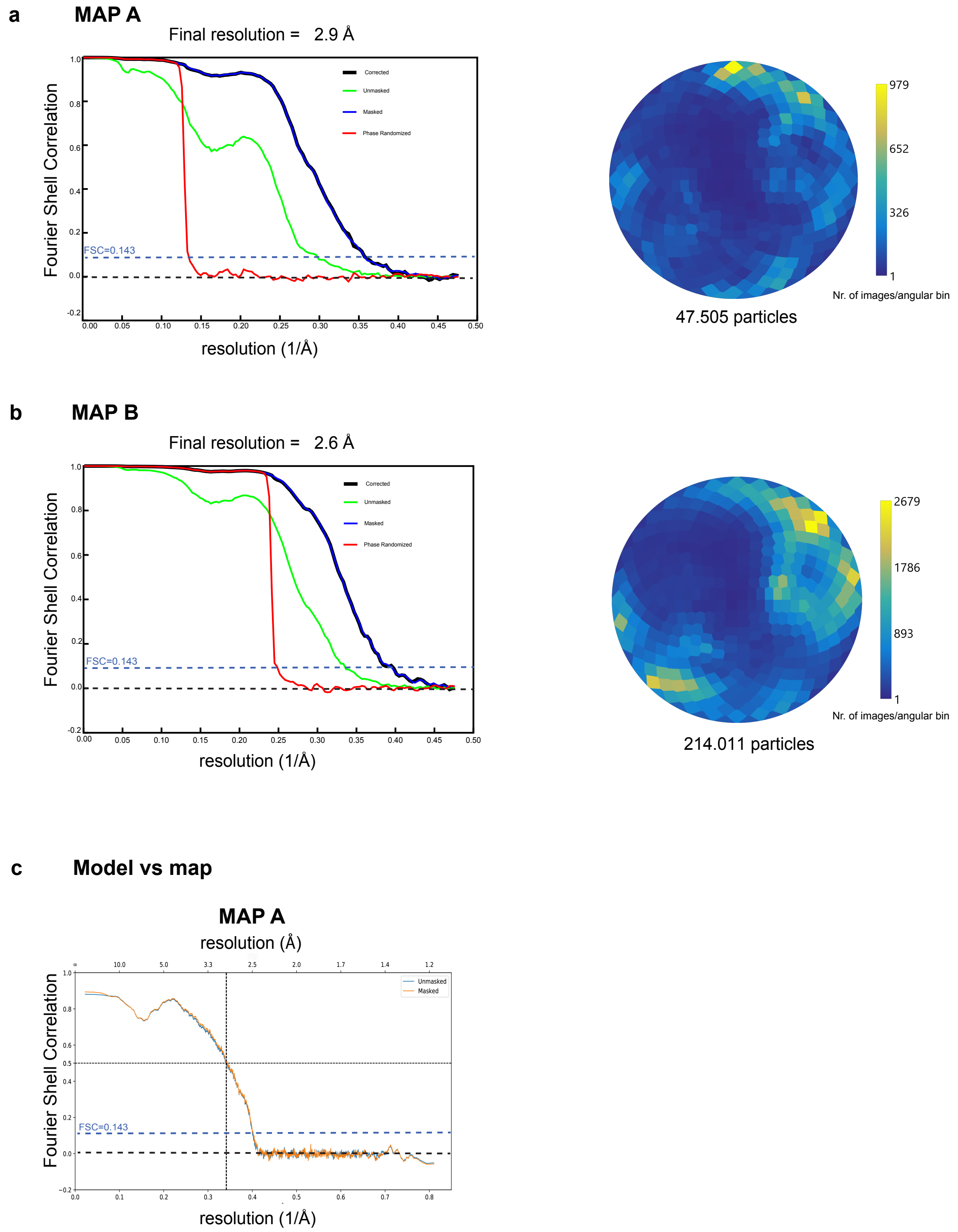

Supplementary Figure 4: Cryo-EM data metrics of Map A and Map B and maps quality calculations

(a) Global resolution of the Map A estimated using the Fourier shell correlation (FSC)=0.143 criterion, and angular distribution plot are shown.

(b) Global resolution of the Map B estimated using the Fourier shell correlation (FSC)=0.143 criterion, and angular distribution plot are shown (calculated in RELION 5.0[4,5]).
(c) Model-to-Map Fit Metric calculated in phenix[7,8] for Map A.

Supplementary Figure 5

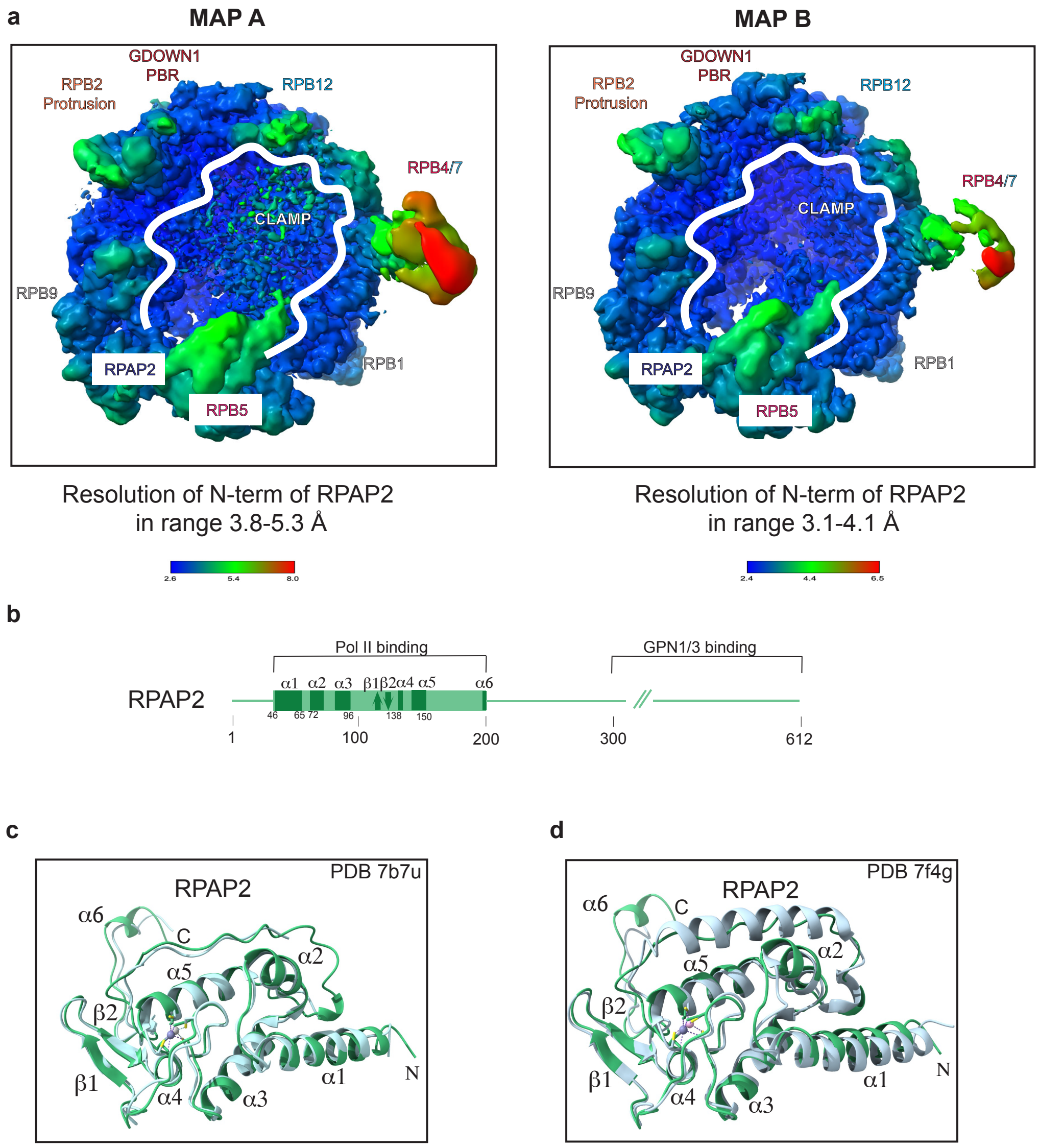

Supplementary Figure 5: Structural details of cfPol II

(a) Local resolution comparison of the N-terminal RPAP2 region (residues 46–197) between Map A (GDOWN1-focused) and Map B (RPAP2-focused). Local resolution maps were calculated in RELION 5.0[4,5] and visualized in ChimeraX[6].
(b) Primary architecture of human RPAP2. The confidently modelled N-terminal region (residues 46-197) is shown in light green boxes with secondary structure elements (α-helices: rectangles; β-sheets: arrows) indicated in dark green. The disordered C-terminal region (residues 198–612) is represented by a green line and could not be modelled in our cryo-EM maps.
(c) Structural alignment of our RPAP2 ribbon model (this study) with published RPAP2 structure PDB 7B7U[9].
(d) Structural alignment of our RPAP2 ribbon model (this study) with published RPAP2 structure PDB 7F4G[10].

Supplementary Figure 6

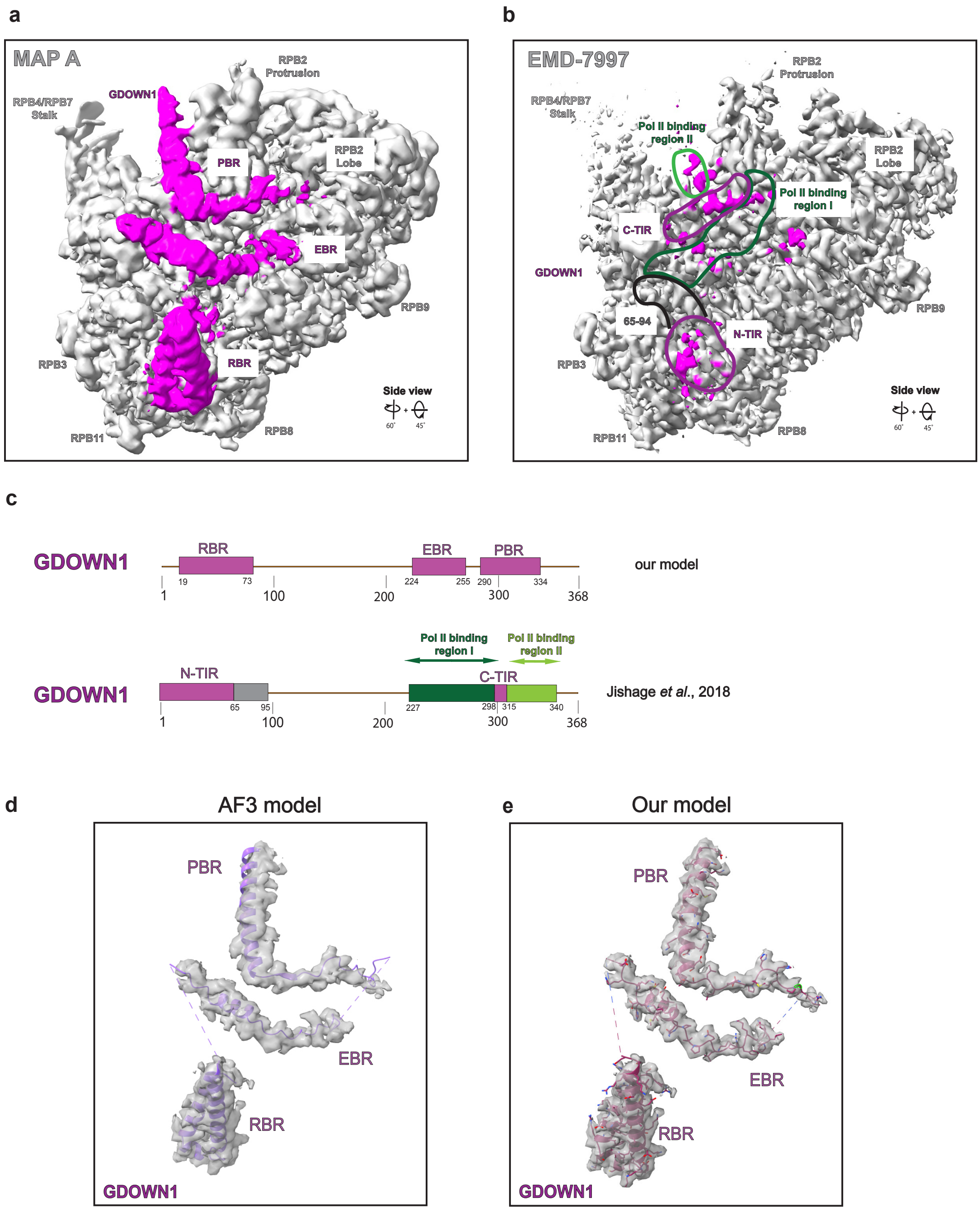

Supplementary Figure 6: Cryo-EM density comparison of GDOWN1 in Map A versus EMDB-3797

(a) *Map A:* GDOWN1 cryo-EM density (magenta) with fitted atomic model from Map A. Regions binding RPB2, RPB10, and RPB3 are labeled according to Fig. 2a,b designations (RBR, EBR, PBR).
(b) *EMDB-3797* (Jishage *et al*., 2018[11]*):* GDOWN1 density (magenta) fitted with the same

atomic model as in (a). Binding regions labeled per Jishage *et al*., 2018[11].
(c) Domain architecture comparison of human GDOWN1 between our model and Jishage *et al.*, 2018[11].
(d) The AlphaFold 3[12] model of GDOWN1 placed in Map A. The GDOWN1 structure was extracted from the AlphaFold 3[12] model of GDOWN1-RPAP2-Pol II complex. The map, in which the structure of GDOWN1 is fitted, was generated from Map A using Chimera X[6] .
(e) The final model of GDOWN1 from our reconstruction placed in MAP A. The GDOWN1 structure was extracted from our final model. The side chains in the structure of GDOWN1 are shown. The map was extracted from the Map A using Chimera X[6] .

Supplementary Figure 7

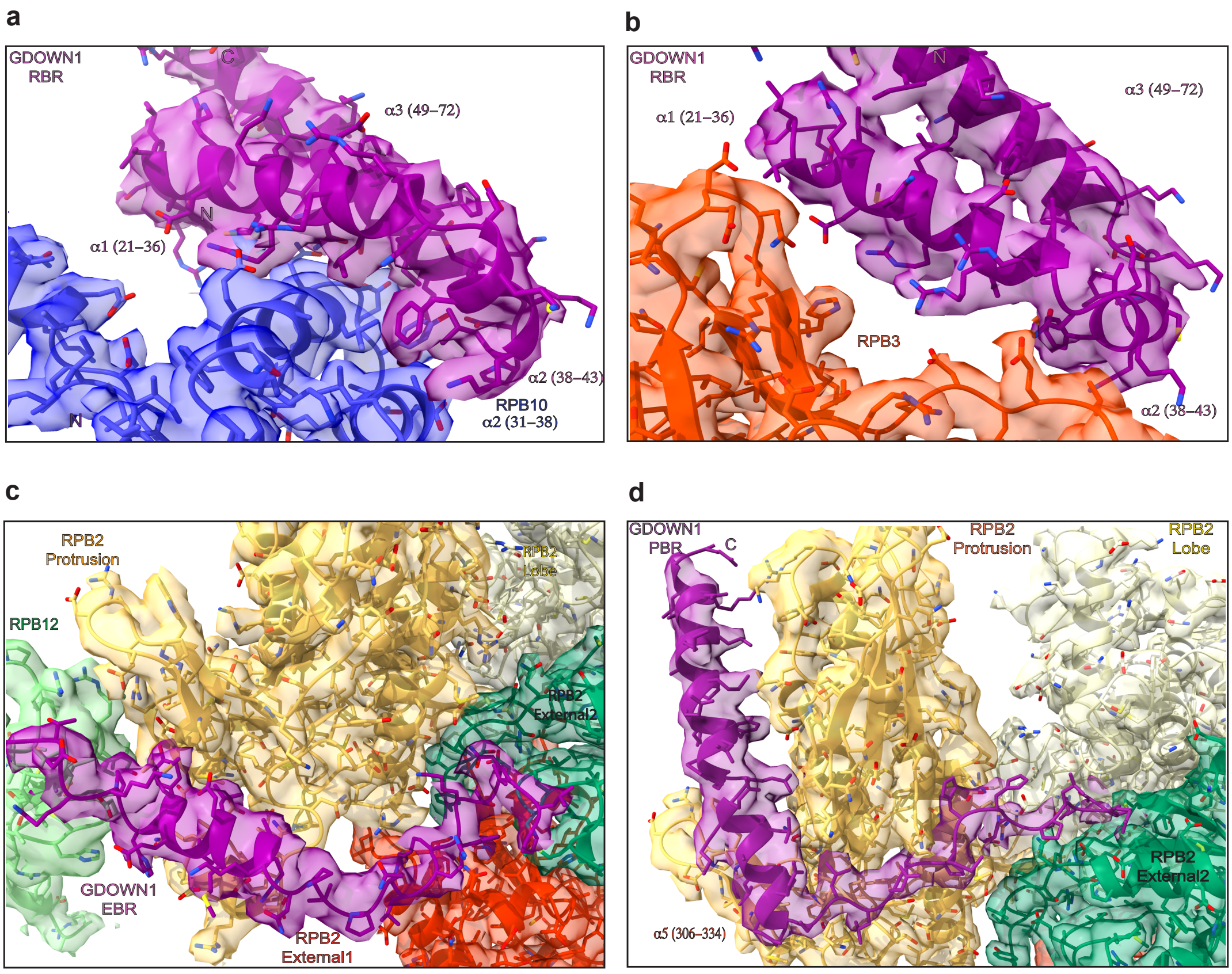

Supplementary Figure 7: GDOWN1 binding interfaces with Pol II subunits placed in the Map A.

(a) Interaction interface between GDOWN1 RBR (residues 19–73) and RPB10.
(b) Interaction interface between GDOWN1 RBR (residues 19–73) and RPB3.
(c) Interaction interface between GDOWN1 EBR (residues 224–255) and RPB2 (protrusion, external 1 and 2 domains).
(d) Interaction interface between GDOWN1 PBR (residues 290–334) and RPB2 (protrusion domain, external domain 2). Color coding matches Fig. 2b.
The corresponding maps from Map A and interaction interfaces from our model were generated using ChimeraX[6].

Supplementary Figure 8

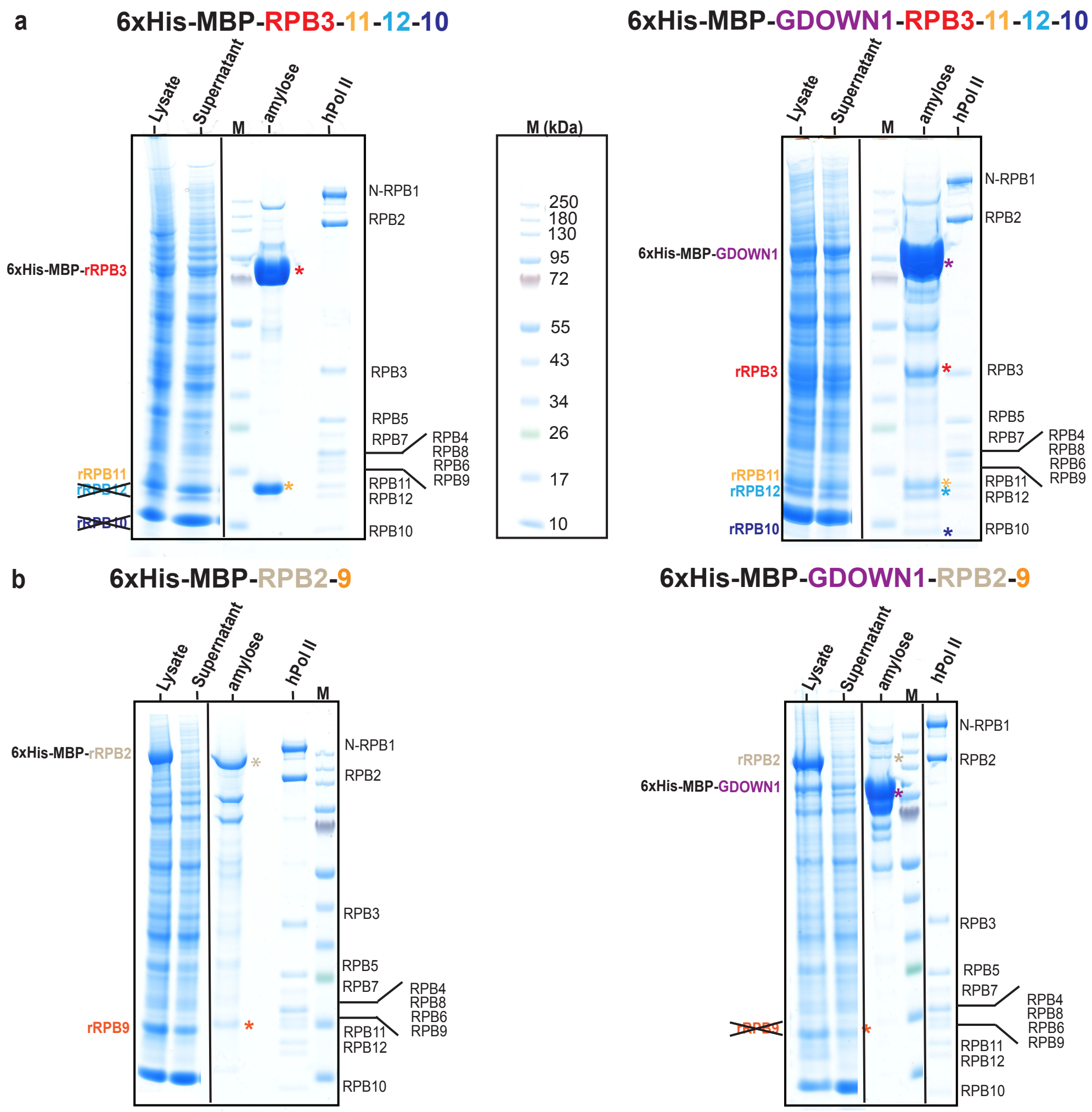

Supplementary Figure 8: SDS-PAGE analysis of interactions between GDOWN1 and the RPB2 and RPB3 subcomplexes

(a) *Left:* Expression of the RPB3 subcomplexes (RPB3-RPB11-RPB12-RPB10) with an N-terminal 6xHis-MBP tagged RPB3 (*Lysis, Supernatant*). *Amylose:* pulldown analysis of the proteins purified through the MBP tag on RPB3 using amylose beads (Methods). M- Protein Marker; hPol II: human nfPol II.
Middle: Molecular weight protein marker (M), in kDa
Right: Co-expression of the RPB3 subcomplexes (RPB3-RPB11-RPB12-RPB10 (without a tag) together with N-terminal 6xHis-MBP tagged GDOWN1 (*Lysis, Supernatant*). *Amylose:* pulldown analysis of the proteins purified through MBP tag on GDOWN1 using amylose beads (Methods). M- Protein Marker; hPol II: isolated human nfPol II
(b) *Left:* Expression of the RPB2 subcomplexes (RPB2-RPB9) with an N-terminal 6xHis-MBP tag on RPB2 (*Lysis, Supernatant*). *Amylose:* pulldown analysis of the proteins purified through MBP tag on RPB2 using amylose beads (Methods). M- Protein Marker; hPol II: human nfPol II

Right: Co-expression of the RPB2 subcomplexes (RPB2-RPB9 without a tag) together with N-terminal 6xHis-MBP tagged GDOWN1 (*Lysis, Supernatant*). *Amylose:* pulldown analysis of the proteins purified through MBP tag on GDOWN1 using amylose beads (Methods). M-Protein Marker; hPol II: isolated human nfPol II. Source data are provided as a Source Data file.

Supplementary Figure 9

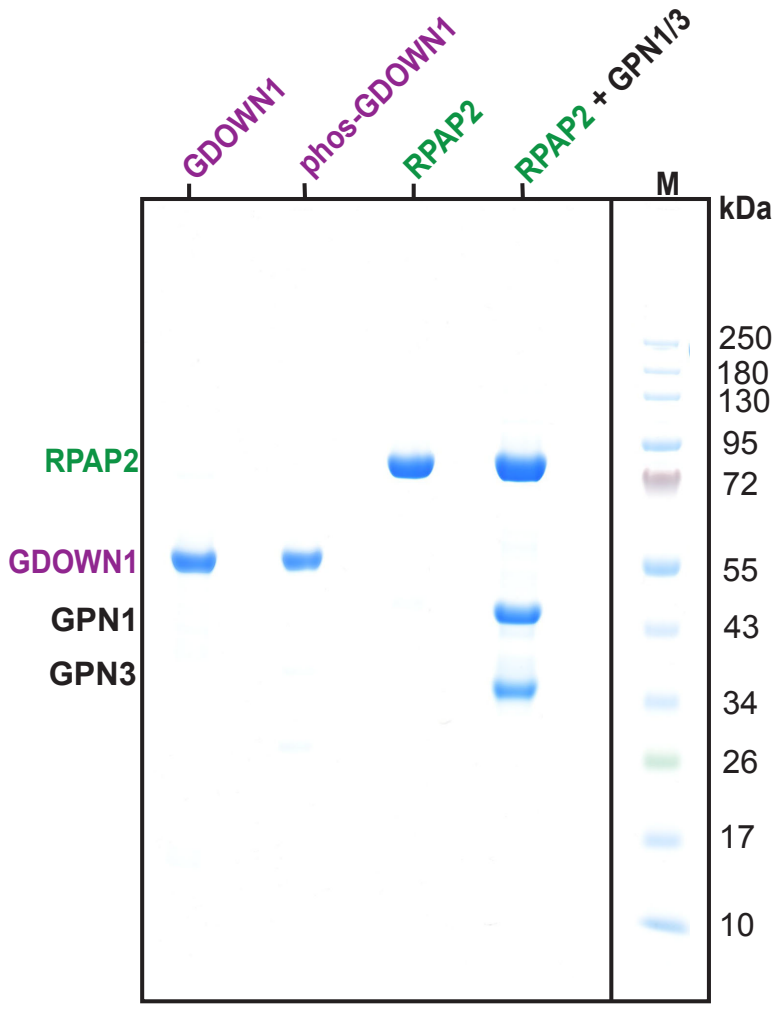

Supplementary Figure 9: SDS-PAGE analysis of the proteins used in *in vitro* transcription initiation assay.

M- Molecular weight protein marker, in kDa. Source data are provided as a Source Data file.

sSupplementary Figure 10

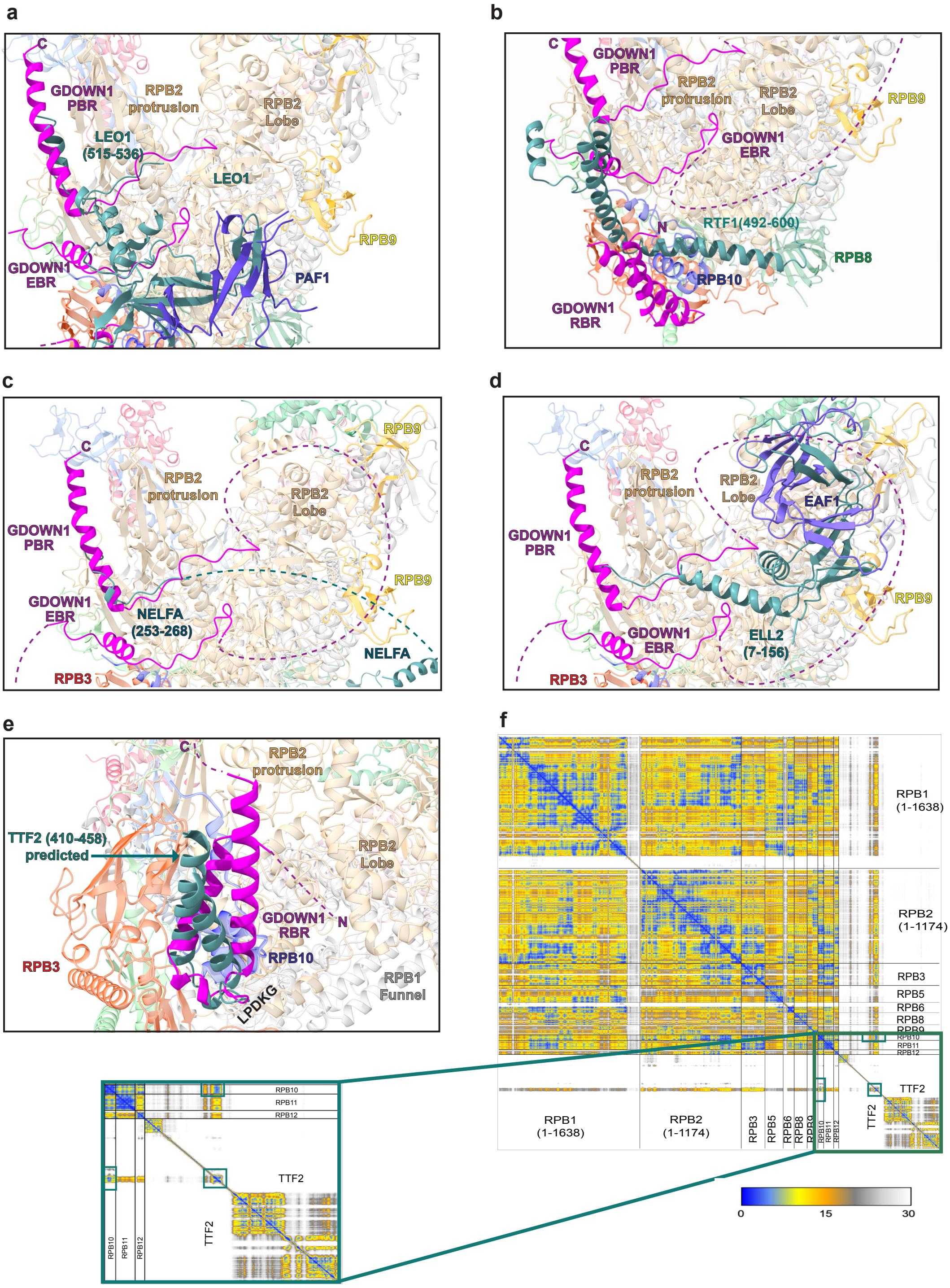

Supplementary Figure 10: Steric clashes between GDOWN1 and transcription elongation and pausing factors, as well as between GDOWN1 and the transcription termination factor TTF2.

(a) GDOWN1 PBR (magenta) sterically clashes with LEO1 (teal; PDB: 9RTT[13])
(b) GDOWN1-RPB2 interface conflicts with RTF1 (teal; PDB: 6TED[14]), an elongation/pausing/chromatin regulator.
(c) GDOWN1 obstructs NELFA tentacle domain binding (teal; PDB: 8UI0[15]).
(d) GDOWN1 PBR/EBR regions block ELL2-EAF1 complex binding (ELL2: teal; EAF1: blue; PDB: 7OKX[16]).
(e) Superposition of TTF2-Pol II AlphaFold 3[12] model reveals clashes between GDOWN1-RBR (RPB10 interface) and TTF2 residues 410–458. Pol II shown at 80% transparency.
(f) *Left:* Predicted Aligned Error (PAE) plot for TTF2-Pol II AlphaFold3[12] model visualized in ChimeraX[6] . Blue boxes indicate TTF2 new fold (residues 410–458) binding RPB10. *Left:* PAE key is shown. Higher PAE values indicate lower structural confidence. The PAE plot's zoom-in box highlights the interaction between TTF2 (aa 410-458) and RPB10. iPTM, pTM and pIDDT were estimated 0.74, 0.77 and between 70-90 %, respectively.

Supplementary Table 1

Protein complex	Total spectra	Sequence coverage (%)	Mw (kDa)
**human Pol II**			
N-term 6xHisTwinStrep-RPB1	9002	77	223
RPB2	6734	91	134
RPB7	1370	100	19
RPB3	1218	94	31
RPB4	1118	80	16
RPB9	973	87	15
RPB5	928	85	25
RPB8	534	91	17
RPB10	517	94	8
RPB11	428	100	13
RPB6	249	55	14.5
RPB12	142	62	7
**Assembly and transport factors**			
RPAP2	1490	80	70
Gdown1	1334	92	42
GPN3	580	68	33
RECQL5	537	76	109
GPN1	496	81	42
RUVB2	224	79	51
RUVB1	214	82	50
HS71A	152	66	70
HSPA8	127	56	71
SLC7A6OS (hIWR1)	58	45	35
URI1	58	44	60
RPAP3	36	41	75.7
HSP90	21	14	84.6
**Cytoplasmic contaminants**			
Acetyl_CoA carboxylase 1	6394	95	266
Acetyl_CoA carboxylase 2	898	63	277
Tubulin cluster Beta	1286	85	50
Tubulin cluster alpha	672	97	50
UBR4	307	33	574
RPS3 (ribosomal protein)	80	81	27
RPS4X (ribosomal protein)	61	52	30
**Mitochondrial contaminants**			
PCCA	247	67	80
MCCB	198	76	61
MCCA	172	62	80.5
PCCB	194	81	58
**Nuclear proteins/complexes**			
INT1	407	54	244
INT4	203	62	108
INT7	183	63	107
INT2	140	49	134
INT3	136	49	118
INT10	135	57	82
INTS13	129	57	80
INT5	113	52	108
INT6	105	46	100
INT8	86	41	113
INT9	84	54	73.8
INT14	60	61	57
SPT6	97	36	199
Med23	64	20	156.5
WRNIP1	137	58	72
FBX21	146	51	70.3
SKP1	68	89	19

Supplementary Table 1: Mass spectrometry analysis of endogenous cfPol II complex

Proteins were identified by LC-MS/MS from BS3 cross-linked sample of endogenous cfPol II. Total spectra, sequence coverage (%), and molecular weight (Mw) are shown. Proteins are grouped by functional class.

Supplementary Table 2

	**cfPol II-GDOWN1-RPAP2 MAP A (EMDB: EMD-56542) (PDB: 28JC)**	**cfPol II-GDOWN1-RPAP2 MAP B (EMDB: EMD-56541)**
**Data collection and processing**		
Magnification	81,000x	81,000x
Voltage (kV)	300	300
Electron exposure (e–/Å2)	39.91	39.91
Defocus range (µm)	0.6-2	0.6-2
Pixel size (Å)	1.05	1.05
Initial particle images (no.)	4.113,941	4.113,941
Final particle images (no.)	47,505	214,011
Symmetry imposed	C1	C1
Map resolution (Å) at FSC = 0.143	2.9	2.6
Map resolution range (Å)	2.9-5.0	2.6-5.0
**Refinement and validation**		
Initial models used (PDB code)	7ozn	
**Model composition**		
Non-hydrogen atoms	29,492	
Protein residues	3,668	
Ligands	Zn: 6	
**Mean B factors ($Å^2$)**		
Protein	90.93	
Ligand	156.63	
**R.m.s. deviations**		
Bond lengths (Å)	0.004	
Bond angles (°)	0.804	
**Validation**		
MolProbity score	1.68	
Clashscore	6.37	
Poor rotamers (%)	0	
**Ramachandran plot**		
Disallowed (%)	0.00	
Allowed (%)	4.76	
Favored (%)	95.24	

Supplementary Table 2: Cryo-EM data acquisition, processing and refinement statistics.

Supplementary References

1 Dignam, J. D., Lebovitz, R. M. & Roeder, R. G. Accurate transcription initiation by RNA polymerase II in a soluble extract from isolated mammalian nuclei. *Nucleic Acids Res* **11**, 1475-1489 (1983). https://doi.org/10.1093/nar/11.5.1475

2 PyMOL Molecular Graphics System. v. 3.1.5.1 (Schrödinger, LLC., 2023).

3 Tegunov, D. & Cramer, P. Real-time cryo-electron microscopy data preprocessing with Warp. *Nature Methods* **16**, 1146-1152 (2019). https://doi.org/10.1038/s41592-019-0580-y

4 Scheres, S. H. A Bayesian view on cryo-EM structure determination. *J Mol Biol* **415**, 406-418 (2012). https://doi.org/10.1016/j.jmb.2011.11.010

5 Zivanov, J. *et al.* New tools for automated high-resolution cryo-EM structure determination in RELION-3. *eLife* **7**, e42166 (2018). https://doi.org/10.7554/eLife.42166

6 Pettersen, E. F. *et al.* UCSF Chimera--a visualization system for exploratory research and analysis. *J Comput Chem* **25**, 1605-1612 (2004). https://doi.org/10.1002/jcc.20084

7 Adams, P. D. *et al.* PHENIX: a comprehensive Python-based system for macromolecular structure solution. *Acta Crystallogr D Biol Crystallogr* **66**, 213-221 (2010). https://doi.org/10.1107/s0907444909052925

8 Afonine, P. V. *et al.* Real-space refinement in PHENIX for cryo-EM and crystallography. *Acta Crystallogr D Struct Biol* **74**, 531-544 (2018). https://doi.org/10.1107/s2059798318006551

9 Fianu, I., Dienemann, C., Aibara, S., Schilbach, S. & Cramer, P. Cryo-EM structure of mammalian RNA polymerase II in complex with human RPAP2. *Communications Biology* **4**, 606 (2021). https://doi.org/10.1038/s42003-021-02088-z

10 Wang, X. *et al.* RPAP2 regulates a transcription initiation checkpoint by inhibiting assembly of pre-initiation complex. *Cell Rep* **39**, 110732 (2022). https://doi.org/10.1016/j.celrep.2022.110732

11 Jishage, M. *et al.* Architecture of Pol II(G) and molecular mechanism of transcription regulation by Gdown1. *Nat Struct Mol Biol* **25**, 859-867 (2018). https://doi.org/10.1038/s41594-018-0118-5

12 Abramson, J. *et al.* Accurate structure prediction of biomolecular interactions with AlphaFold 3. *Nature* **630**, 493-500 (2024). https://doi.org/10.1038/s41586-024-07487-w

13 Zheenbekova, A. *et al.* IWS1 positions downstream DNA to globally stimulate Pol II elongation. *Nat Commun* **16**, 7747 (2025). https://doi.org/10.1038/s41467-025-62913-5

14 Vos, S. M., Farnung, L., Linden, A., Urlaub, H. & Cramer, P. Structure of complete Pol II–DSIF–PAF–SPT6 transcription complex reveals RTF1 allosteric activation. *Nature Structural & Molecular Biology* **27**, 668-677 (2020). https://doi.org/10.1038/s41594-020-0437-1

15 Su, B. G. & Vos, S. M. Distinct negative elongation factor conformations regulate RNA polymerase II promoter-proximal pausing. *Mol Cell* **84**, 1243-1256.e1245 (2024). https://doi.org/10.1016/j.molcel.2024.01.023

16 Chen, Y. *et al.* Allosteric transcription stimulation by RNA polymerase II super elongation complex. *Molecular Cell* **81**, 3386-3399.e3310 (2021). https://doi.org/10.1016/j.molcel.2021.06.019
