# Supplementary material for: Structure of human cytoplasmic Pol II complex explains global transcription repression by GDOWN1
Source: Nat Commun. 2026 Aug 5;17:7854. doi: 10.1038/s41467-026-76334-5 (PMC13443923; doi:10.1038/s41467-026-76334-5)
Supplement: Supplementary file 2 [file 41467_2026_76334_MOESM2_ESM.pdf]

Structure of human cytoplasmic Pol II complex explains global transcription repression by GDOWN1

Jana Schmitzová[1], Yumeng Zhan[1], Srinivasan Rengachari[1,2], Frauke Grabbe[1], Olexandr Dybkov[3], Henning Urlaub[3,4], Michael Lidschreiber[1], Christian Dienemann[1,‡,*], Patrick Cramer[1,*]

[1] Department of Molecular Biology, Max Planck Institute for Multidisciplinary Sciences, Göttingen, Germany.
[2] Current address: Section for Structural and Synthetic Biology, Department of Infectious Disease, Imperial College London, London, United Kingdom.
[3] Research Group Bioanalytical Mass Spectrometry, Max Planck Institute for Multidisciplinary Sciences, Göttingen, Germany.
[4] Bioanalytics, Department of Clinical Chemistry, University Medical Center, Göttingen, Germany.
[‡]Lead contact
[*] Correspondence: christian.dienemann@mpinat.mpg.de and patrick.cramer@mpinat.mpg.de

Description of Additional Supplementary Files:

File Name: Supplementary Data 1

Description: Protein-protein crosslinks identified in the human cfPol II complex (dataset 1). BS3-crosslinks were identified using pLink 2.3.11 and filtered at FDR of either 1 or 5%. Crosslinks supported by less than 2 crosslinked peptide-spectrum matches (CSMs) were excluded. The number of CSMs and the highest score are indicated for each crosslinked peptide. "Residue 1" and "Residue 2" are the crosslinked residue pairs in Protein 1 and Protein 2, respectively.

File Name: Supplementary Data 2

Description: Protein-protein crosslinks identified in the cf Pol II complex (dataset 1) as well as in the cfPol II with spiked-in assembly factors GDOWN1, GPN1, GPN3 and RPAP2 (dataset 2). BS3-crosslinks were identified using pLink 3.0.17 and filtered at peptide-pair level FDR of either 1 or 5%. The number of CSMs and minimal E-Values are indicated for each crosslinked peptide. "Residue 1" and "Residue 2" are the crosslinked residue pairs in Protein 1 and Protein 2, respectively.
