# Supplementary material for: Structure of human cytoplasmic Pol II complex explains global transcription repression by GDOWN1
Source: Nat Commun. 2026 Aug 5;17:7854. doi: 10.1038/s41467-026-76334-5 (PMC13443923; doi:10.1038/s41467-026-76334-5)
Supplement: Supplementary file 3 [file 41467_2026_76334_MOESM5_ESM.pdf]

nature portfolio

Corresponding author(s): Prof.Dr.Patrick Cramer
Dr. Christian Dieneman

Last updated by author(s): 29.06. 2026

Reporting Summary

Nature Portfolio wishes to improve the reproducibility of the work that we publish. This form provides structure for consistency and transparency in reporting. For further information on Nature Portfolio policies, see our Editorial Policies and the Editorial Policy Checklist.

Statistics

For all statistical analyses, confirm that the following items are present in the figure legend, table legend, main text, or Methods section.

n/a	Confirmed	
☐	☒	The exact sample size (n) for each experimental group/condition, given as a discrete number and unit of measurement
☐	☒	A statement on whether measurements were taken from distinct samples or whether the same sample was measured repeatedly
☒	☐	The statistical test(s) used AND whether they are one- or two-sided<br>*Only common tests should be described solely by name; describe more complex techniques in the Methods section.*
☒	☐	A description of all covariates tested
☒	☐	A description of any assumptions or corrections, such as tests of normality and adjustment for multiple comparisons
☐	☒	A full description of the statistical parameters including central tendency (e.g. means) or other basic estimates (e.g. regression coefficient) AND variation (e.g. standard deviation) or associated estimates of uncertainty (e.g. confidence intervals)
☒	☐	For null hypothesis testing, the test statistic (e.g. F, t, r) with confidence intervals, effect sizes, degrees of freedom and P value noted<br>*Give P values as exact values whenever suitable.*
☒	☐	For Bayesian analysis, information on the choice of priors and Markov chain Monte Carlo settings
☒	☐	For hierarchical and complex designs, identification of the appropriate level for tests and full reporting of outcomes
☒	☐	Estimates of effect sizes (e.g. Cohen's d, Pearson's r), indicating how they were calculated

Our web collection on statistics for biologists contains articles on many of the points above.

Software and code

Policy information about availability of computer code

Data collection	serialEM v4.0, uHPLC-ESI-MS/MS analysis
Data analysis	CryoSPARC v4.7.1, Relion 5.0, ChimeraX v1.10, PHENIX v1.20.1, WARP v1.0.9, Coot 0.9.8.92, AlphaFold 3, pLink2.3.11, pLink3.0.17 , MASCOT v.2.3.02, PyMol 3.1.5.1

For manuscripts utilizing custom algorithms or software that are central to the research but not yet described in published literature, software must be made available to editors and reviewers. We strongly encourage code deposition in a community repository (e.g. GitHub). See the Nature Portfolio guidelines for submitting code & software for further information.

Data

Policy information about availability of data

All manuscripts must include a data availability statement. This statement should provide the following information, where applicable:

- Accession codes, unique identifiers, or web links for publicly available datasets
- A description of any restrictions on data availability
- For clinical datasets or third party data, please ensure that the statement adheres to our policy

The cryo-EM reconstructions and final model have been deposited in the Electron Microscopy Data Base (EMDB) under accession codes for Map A: EMD-56542: https://www.ebi.ac.uk/emdb/EMD-56542, and for Map B: EMD-56541: https://www.ebi.ac.uk/emdb/EMD-56541,and in the Protein Data Bank (PDB) under

accession code 28JC: https://www.rcsb.org/structure/unreleased/28JC . Supplementary information and Source data are provided with this paper.
Input models used are: 7OZN and AlphaFold 3 model for Gdown1 and RPAP2 bound to human Pol II;

Research involving human participants, their data, or biological material

Policy information about studies with human participants or human data. See also policy information about sex, gender (identity/presentation), and sexual orientation and race, ethnicity and racism.

Reporting on sex and gender	not applicable
Reporting on race, ethnicity, or other socially relevant groupings	not applicable
Population characteristics	not applicable
Recruitment	not applicable
Ethics oversight	not applicable

Note that full information on the approval of the study protocol must also be provided in the manuscript.

Field-specific reporting

Please select the one below that is the best fit for your research. If you are not sure, read the appropriate sections before making your selection.

☒ Life sciences ☐ Behavioural & social sciences ☐ Ecological, evolutionary & environmental sciences

For a reference copy of the document with all sections, see nature.com/documents/nr-reporting-summary-flat.pdf

Life sciences study design

All studies must disclose on these points even when the disclosure is negative.

Sample size	CryoEM: No sample size calculations were made and no statistical methods were used. Cryo-EM data were collected on a grid prepared from one in vitro reconstituted sample. This is a standard practice in cryo-EM.<br>XL-MS: cfPol II was prepared twice from 8L of 3–6 × 10^6 cells/mL HEK 293 cells and crosslinked in 3 independent experiments
Data exclusions	CryoEM: Particle classes exhibiting denaturation artifacts, low-quality alignments, or poor structural features were systematically excluded during processing. This standard cryo-EM practice minimizes the noise in reconstruction by eliminating non-informative particles.<br>XL-MS:no data exclusion. The pLink results were filtered at false discovery rate. (FDR) of either 1 or 5% at spectral level (pLink 2.3.11) or peptide-pair level (pLink 3.0.17) and presented in Supplementary Tables 1 and 2, respectively.
Replication	In vitro transcription initiation assay is repeated three times (Fig4ab,c) and four times (Fig4e,d). The results are reproducible.<br>Cryo-EM structure determination was not replicated. This is a standard practice in cryo-EM. Proteins were prepared using established protocol, which normally yield enough material for several reproducible structural and biochemical experiments.<br>XL-MS: Total 3 crosslinking MS experiments were performed with different concentration of BS3 (0.7, 1.4 and 2 mM) using two independent preparations of cfPol II. uHPLC-ESI-MS data were acquired with 3 or 4 injection replicates.
Randomization	CryoEM: Resolution estimation was performed by the gold-standard FSC of two randomly assigned halves of the data set.<br>XL-MS: No randomization was performed. Not relevant for this study.
Blinding	not applicable

Reporting for specific materials, systems and methods

We require information from authors about some types of materials, experimental systems and methods used in many studies. Here, indicate whether each material, system or method listed is relevant to your study. If you are not sure if a list item applies to your research, read the appropriate section before selecting a response.

Materials & experimental systems

n/a	Involved in the study	
☐	☒	Antibodies
☐	☒	Eukaryotic cell lines
☒	☐	Palaeontology and archaeology
☒	☐	Animals and other organisms
☒	☐	Clinical data
☒	☐	Dual use research of concern
☒	☐	Plants

Methods

n/a	Involved in the study	
☒	☐	ChIP-seq
☒	☐	Flow cytometry
☒	☐	MRI-based neuroimaging

Antibodies

Antibodies used

antibody against Twin Strep (ab76949, Fischer Scientific), Used at a dilution of 1:1,000 for Western blotting

Validation

The rabbit polyclonal primary antibody against Twin Strep was validated for use in Western blotting through the detection of the Twin Strep tag in the RPB1 subunit of Pol II.
From manufacturer: Anti-Strep-tag II antibody (ab76949) is a rabbit polyclonal antibody and is validated for use in WB in human, mouse,rat, Saccharomyces cerevisiae samples.
Anti-Strep-tag II antibody (ab76949) has been cited over 57 times in peer reviewed journals and is trusted by the scientific community.

Eukaryotic cell lines

Policy information about cell lines and Sex and Gender in Research

Cell line source(s)

Hi5 cells: Trichoplusia ni insect cells (BTI-Tn-5B1-4) in ESF921 medium, Expression Systems: Item 96-001-01; Sf9 cells, from the parental Spodoptera frugiperda cell line (IPLB-Sf21-AE): Sf9 cells in ESF921 medium, Expression Systems: Item 96-001-01; Sf21 cells, from ovarian Spodoptera frugiperda cell line (IPLB-Sf21-AE): Sf21 insect cells in ESF921 medium, Expression Systems: Item 96-001-01;
Hek293 (ATCC CRL-1573) cell line in medium: HEKONE/S, Capricorn Cat.Nr. HEKS-1000ML, Stable Glutamin (200mM) Capricorn Cat.Nr.STA-B, Penicillin-Streptomycin Sigma P0781-100ML;

Authentication

Insect cells were provided by supplier; Hek293 clone C1N1 with N-terminal tagged RPB1 was prepared in our laboratory by CRISPR/Cas9 genome editing: original Hek293 (ATCC CRL-1573) cell line was purchased from ATCC

Mycoplasma contamination

HEK293 C1N1 cells were confirmed mycoplasma-free; insect cells were not tested

Commonly misidentified lines
(See ICLAC register)

No commonly misidentified cell line was used

Plants

Seed stocks

not applicable

Novel plant genotypes

not applicable

Authentication

not applicable
