# Supplementary material for: Structure of human cytoplasmic Pol II complex explains global transcription repression by GDOWN1
Source: Nat Commun. 2026 Aug 5;17:7854. doi: 10.1038/s41467-026-76334-5 (PMC13443923; doi:10.1038/s41467-026-76334-5)
Supplement: Supplementary file 4 [file 41467_2026_76334_MOESM6_ESM.pdf]

nature portfolio

Peer Review File

Structure of human cytoplasmic Pol II complex explains global transcription repression by GDOWN1

Corresponding Author: Dr Christian Dienemann

This file contains all reviewer reports in order by version, followed by all author rebuttals in order by version.

Version 0:

Reviewer comments:

Reviewer #1

(Remarks to the Author)
RNA polymerase (Pol II) is a large multi-protein complex that synthesises a variety of RNA types in eukaryotic cells. Because of its key roles in cellular function, it has been intensively studied by many labs, leading to detailed mechanistic insight and a plethora of high-resolution structures that illuminate various stages of Pol II transcription. In contrast, structural data on how this giant molecular machine is assembled are comparably scarce. In their submission, Schmitzova et al. investigate a late cytoplasmic Pol II assembly intermediate bound by Gdown1 and other assembly factors. From their structural data, the authors derive explanations for the known functions of Gdown1 in repression of Pol II transcription when nuclear Gdown1 levels are high.

The paper is well written and the rationale is sound. The structural work is generally of high quality (though more information is needed, see below), while the functional interpretation relies heavily on prior structural work and literature data. However, some weaknesses and omissions need to be addressed before a decision on the suitability of the manuscript for Nature Communications can be made.

Main issue:

The validation information for the cryo-EM structure is incomplete. The manuscript does not include half-map FSC curve, model vs. map FSC curve, or information on map anisotropy (e.g. 3D FSC). These are integral part of any cryo-EM paper (as the authors are certainly aware) and must be added to a revised version of this paper.

Minor queries and suggestions:

Page 3, line 27: RBP1 should be RPB1.

Page 4, line 9: "is previously observed before" -> "was observed previously"

Page 4, line 25: Density views with the Alphafold prediction and the refined model should be added to confirm accurate assignment of the Gdown1 sequence register in the structure (particularly considering the stated deviations from prior studies, page 5 line 19).

Page 6, line 13 and subsequent paragraph: The authors find that cytoplasmic Pol II is unable to initiate transcription and attribute this to the presence of Gdown1. While this inference is consistent with the remaining data and previously published results, it is left unclear if cytoplasmid Pol II would be able to initiate transcription if Gdown1 were released, or if addition of purified Gdown1 to nuclear Pol II would reduce the initiation levels to those of cytoplasmic Pol II. The experiment thus does not answer whether the presence of Gdown1 is the only reason why cytoplasmic Pol II does not initiate. This leaves room for additional experimentation (or use of literature data) to directly address the mechanistic implication derived from the data currently contained in the manuscript.

Page 7, line 3ff: The authors propose that Gdown1 stabilises a Pol II subassembly with RBP2/3. However, given that the different contact sites of Gdown1 on Pol II are separated by flexible linkers, it is not immediately obvious how this would happen. What is the authors' thinking on this mechanism? Do they think that Gdown1 tethers smaller subassemblies to

increase local concentration and facilitate their joining into larger assemblies?

Page 7, 23: "Substantiate" rather than "substantiates".

Figures:

Fig. 2: Where side chains forming interactions are shown, equivalent views shown within the cryo-EM map should be shown in the supplement to substantiate these interactions.

Extended Data Fig. 2, 3, 4: Local resolution visualisations: The use of red for highly resolved regions and blue for low-resolution regions is counterintuitive. While field has not established a clear consensus for a colour scheme for these renderings, it might be more intuitive to use blue for the highly-resolved regions, in analogy to blue being used for low b-factors in X-ray crystallography. The use of a more suitable colour gradient should be considered (e.g. https://www.nature.com/articles/s41467-020-19160-7).

Reviewer #2

(Remarks to the Author)
I co-reviewed this manuscript with one of the reviewers who provided the listed reports. This is part of the Nature Communications initiative to facilitate training in peer review and to provide appropriate recognition for Early Career Researchers who co-review manuscripts.

Reviewer #3

(Remarks to the Author)
"Structure of human cytoplasmic Pol II complex explains global transcription repression by Gdown1"

In this study, the authors present the CryoEM structure of endogenously purified human Pol II bound by the RPAP2-Gdown1 assembly factors, allowing for the rationalization of the mechanism of Gdown1-mediated transcription inhibition of Pol II. This paper is timely, easy to follow and well-written. We would recommend this paper for publication after the following points are addressed:

1. The XL-MS dataset appears sparse (derived from only 75 pmol of crosslinked material without SEC purification), which likely limits sensitivity and coverage. GPN1–3 appear inefficiently crosslinked (Extended Data Fig. 1e). The authors may consider: (i) repeating the XL-MS experiment starting from a larger amount of material and including a size-exclusion step prior to crosslinking; (ii) increasing crosslinker concentration to capture transient GPN interactions.

2. The presentation of the XL-MS data should also include a mapping of crosslinks to the resolved structure with a histogram showing the range of Ca-Ca crosslink distances observed with a violation rate to determine if both datasets are consistent. Additionally, it is unclear which FDR (1 or 5%) was used in the pLINK analysis of the XL-MS data. Please confirm which one was used for the dataset presented.

3. For the assessment of the CryoEM data, please include an FSC, Map vs. Model FSC, viewing angle distribution (or 3DFSC) and a figure showing the fitting of the model into the solved density. Also, in the extended data figure 2, please enlarge the 2D class averages as they are currently difficult to interpret.

4. To demonstrate the steric mechanism of Gdown1 inhibition of transcription, the authors compare their structure to the TTF2-Pol II Alphafold3 prediction. Please include a pLDDT, iPTM and PTM scores to assess the quality of the prediction. The included PAE plot is difficult to understand, perhaps the inclusion of a zoomed panel or enlargement of the panel to allow for a clearer demonstration of the predicted interface?

Further to these points, additional minor points regarding the manuscript should be addressed:

5. The legend for figure 3b does not correspond to the figure.

6. In figure 3d, the authors use a transcription activity assay to demonstrate the Gdown1 inhibition of Pol II transcription. However, for quantification authors should include a densitometric analysis across replicates with a statistical significance of comparison.

7. Extended Data Fig. 1b-c-d: some panels are missing molecular weight markers.

8. For Extended Data Fig. 6f the boxes on the PAE plot are blue instead of red.

Reviewer #4

(Remarks to the Author)
This manuscript by Schmitzova et al describes cryo-EM and mass spectrometry approaches to study the cytoplasmic form of RNA polymerase II (cfPolII) prior to assembly and import into the nucleus. The authors fractionated lysate from a human cell

line to isolate a cytoplasmic complex using an affinity tag on the PolII subunit RPB1. In addition to cfPolII, further proteins co-purified, which have been implicated in PolII assembly (Gdown1, RPAP2, and the GPN1-GPN3 heterodimer). With respect to Gdown1, the manuscript comes to similar conclusions as a recent bioRxiv pre-print from the lab of Carrie Bernecky, which is reassuring. But here's where the story ends and to me the present manuscript feels very much like a preliminary draft and lacks the complementary biochemical data (and/or additional structural data) present in the bioRxiv preprint from the Bernecky lab. In the current form I am not in favor of publication.

The abstract reads like a press release and not like a scientific abstract. I'd suggest using more neutral, objective language to let the data's significance speak for itself.
For example, how do the binding interfaces of Gdown1 and PolII indicate a stabilization of PolII assembly intermediates – the same interface was seen with fully assembled nuclear PolII and the authors themselves say they isolated fully assembled PolII (and not an intermediate) so we cannot know what the assembly intermediate may look like and this statement is a hyperbole.
Overlap of Gdown1 and TFIIB and TFIIF have been reported before by the Roeder lab and others and it is not a new finding.
As far as I can see, there is no model to propose Gdown1's role in assembly, import, and transcription regulation – i.e. a model of Gdown1 implicated in these three independent processes – another exaggeration in my view.

Is the clamp domain visible in low-pass filtered maps? Is it disordered or oscillating? In the latter case, have the authors tried focused 3D classification - please comment on this in the paper.

The authors show focused classification for the RPAP2 density (Extended Fig 3) but we learn next to nothing about it in the manuscript.

Page 4, line 33 (and page 5, line 27): "…first atomic model of Gdown1" feels like hyperbole – about a third of the protein could be modeled.

Page 6, line17-19: This sentence does not make any sense.

The model presented in the Discussion is highly speculative and almost exclusively based on data published in other studies.

Fig. 3: The legend does not match the figure. There's no marker in the left panel of 3c

Extended Data Fig. 6d and 6f: Colors described in legend do not match figure.

Extended Data Table 2: initial particle images number is too low. Are there really 0% Rotamer outliers?

Please provide FSC plots for the final reconstructions.

Version 1:

Reviewer comments:

Reviewer #1

(Remarks to the Author)
The authors have suitably addressed our comments and concerns. The paper is now suitable for publication in Nature Communications, conditional on a few remaining comments (below) being addressed.

Remaining comments:

Fig. 4b (figure legend): Specify whether triplicate experiments were performed as biological or technical replicates.

Extended Data Fig. 7: The authors should consider showing all side chains, rather than a select few, to support their register and contact surface assignment. Are all the side chains implicated in polar interactions actually within distances suitable for hydrogen bonding or formation of salt bridges?

Extended Data Fig. 8: Why did the authors elect to swap the position of the tag for their co-purification assay with/without GDOWN1? The position of the tag alone could affect protein stability or complex formation.

Data integrity: Extended Data Fig. 8 contains multiple instances of apparent lane splicing that are not indicated. If this observation is accurate, this must be corrected.

Reviewer #2

(Remarks to the Author)
I co-reviewed this manuscript with one of the reviewers who provided the listed reports. This is part of the Nature

Communications initiative to facilitate training in peer review and to provide appropriate recognition for Early Career Researchers who co-review manuscripts.

Reviewer #3

(Remarks to the Author)
The authors have clarified all the outstanding issues and the manuscript can be published in its current form

Reviewer #4

(Remarks to the Author)
Thank you for sending the revised manuscript. The authors were able to address my concerns and I am in favor of publication.

Open Access This Peer Review File is licensed under a Creative Commons Attribution 4.0 International License, which permits use, sharing, adaptation, distribution and reproduction in any medium or format, as long as you give appropriate credit to the original author(s) and the source, provide a link to the Creative Commons license, and indicate if changes were made.
In cases where reviewers are anonymous, credit should be given to 'Anonymous Referee' and the source.
The images or other third party material in this Peer Review File are included in the article's Creative Commons license, unless indicated otherwise in a credit line to the material. If material is not included in the article's Creative Commons license and your intended use is not permitted by statutory regulation or exceeds the permitted use, you will need to obtain permission directly from the copyright holder.
To view a copy of this license, visit https://creativecommons.org/licenses/by/4.0/

Responses

Our responses are shown in blue.

Reviewer #1 (Remarks to the Author):

RNA polymerase (Pol II) is a large multi-protein complex that synthesises a variety of RNA types in eukaryotic cells. Because of its key roles in cellular function, it has been intensively studied by many labs, leading to detailed mechanistic insight and a plethora of high-resolution structures that illuminate various stages of Pol II transcription. In contrast, structural data on how this giant molecular machine is assembled are comparably scarce. In their submission, Schmitzova et al. investigate a late cytoplasmic Pol II assembly intermediate bound by Gdown1 and other assembly factors. From their structural data, the authors derive explanations for the known functions of Gdown1 in repression of Pol II transcription when nuclear Gdown1 levels are high.

The paper is well written and the rationale is sound. The structural work is generally of high quality (though more information is needed, see below), while the functional interpretation relies heavily on prior structural work and literature data. However, some weaknesses and omissions need to be addressed before a decision on the suitability of the manuscript for Nature Communications can be made.

Main issue:

The validation information for the cryo-EM structure is incomplete. The manuscript does not include half-map FSC curve, model vs. map FSC curve, or information on map anisotropy (e.g. 3D FSC). These are integral part of any cryo-EM paper (as the authors are certainly aware) and must be added to a revised version of this paper.

We thank the reviewer for the feedback and we now include FSC plots, angular distribution, and model vs map plots for both cryo-EM maps (ED Figure 4).

Minor queries and suggestions:

Page 3, line 27: RBP1 should be RPB1.

We thank the reviewer for spotting this, we corrected it.

Page 4, line 9: "is previously observed before" -> "was observed previously"

We thank the reviewer for spotting this, we corrected it.

Page 4, line 25: Density views with the Alphafold prediction and the refined model should be added to confirm accurate assignment of the Gdown1 sequence register in the structure (particularly considering the stated deviations from prior studies, page 5 line 19).

We thank the reviewer for the suggestion and have now included two ED Figure panels showing the initial AlphaFold 3 model with the cryo-EM density as well as the final model in the density (ED Figure 6e,d). Furthermore, we now also include ED Figure panels showing the structural details of the GDOWN1-Pol II interactions including the EM density (ED Figure 7).

Page 6, line 13 and subsequent paragraph: The authors find that cytoplasmic Pol II is unable to initiate transcription and attribute this to the presence of Gdown1. While this inference is consistent with the remaining data and previously published results, it is left unclear if cytoplasmid Pol II would be able to initiate transcription if Gdown1 were released, or if addition of purified Gdown1 to nuclear Pol II would reduce the initiation levels to those of cytoplasmic Pol II. The experiment thus does not answer whether the presence of Gdown1 is the only reason why cytoplasmic Pol II does not initiate. This leaves room for additional experimentation (or use of literature data) to directly address the mechanistic implication derived from the data currently contained in the manuscript.

We thank the reviewer for the suggestion and now include more detailed biochemistry to disentangle the contribution of the cfPol II components to transcription inhibition. In particular we performed the transcription initiation assays using nuclear human Pol II and added recombinant GDOWN1, RPAP2, and RPAP2-GPN1-GPN3 complex to it. Our results show that RPAP2 reduces transcription initiation by only 40% while GDOWN1 reduces initiation by 80%. Both together abolish transcription initiation in our assay. We therefore conclude that both factors are important for the inhibition, however, GDOWN1 contributes the majority of the inhibitory effect. We have changed the results and discussion accordingly.

Page 7, line 3ff: The authors propose that Gdown1 stabilises a Pol II subassembly with RBP2/3. However, given that the different contact sites of Gdown1 on Pol II are separated by flexible linkers, it is not immediately obvious how this would happen. What is the authors' thinking on this mechanism? Do they think that Gdown1 tethers smaller subassemblies to increase local concentration and facilitate their joining into larger assemblies?

We thank the reviewer for this question. Assembly and folding of large complexes such as Pol II are a difficult task for the cell and require many chaperones and co-factors *in vivo*. GDOWN1 shows an intricate interaction network spanning across many Pol II

subunits (RPB2-3-9-10-11-12). We think that GDOWN1 either helps to stabilize subassemblies comprising these subunits by solubilizing subunits and/or bringing them together in close proximity during assembly of Pol II. We have now performed co-expression experiments that show that GDOWN1 facilitates soluble expression of a subassembly containing RPB3-10-11-12. This is in line with the molecular interactions we observe in the structure and supports the hypothesis that GDOWN1 may help to stabilize this subassembly during Pol II assembly *in vivo*. In contrast, we could not observe a similar effect on soluble expression of the RPB2-RPB9 subassembly. In summary, we have now clarified our idea of GDOWN1 as an assembly factor in the context of the RPB3-10-11-12 subassembly and also made this clearer in the text.

Page 7, 23: "Substantiate" rather than "substantiates".

We thank the reviewer for spotting this, we corrected it.

Figures:

Fig. 2: Where side chains forming interactions are shown, equivalent views shown within the cryo-EM map should be shown in the supplement to substantiate these interactions.

We now also include ED Figure panels showing the structural details of the GDOWN1-Pol II interactions including the EM density (ED Figure 7).

Extended Data Fig. 2, 3, 4: Local resolution visualisations: The use of red for highly resolved regions and blue for low-resolution regions is counterintuitive. While field has not established a clear consensus for a colour scheme for these renderings, it might be more intuitive to use blue for the highly-resolved regions, in analogy to blue being used for low b-factors in X-ray crystallography. The use of a more suitable colour gradient should be considered (e.g. https://www.nature.com/articles/s41467-020-19160-7).

We thank the reviewer for the suggestion (and the useful pointer to that paper). We have now changed the color scales of the local resolution plots accordingly (i.e. blue-green-red for high-medium-lower resolution).

Reviewer #3 (Remarks to the Author):

“Structure of human cytoplasmic Pol II complex explains global transcription repression by Gdown1”

In this study, the authors present the CryoEM structure of endogenously purified human

Pol II bound by the RPAP2-Gdown1 assembly factors, allowing for the rationalization of the mechanism of Gdown1-mediated transcription inhibition of Pol II. This paper is timely, easy to follow and well-written. We would recommend this paper for publication after the following points are addressed:

1. The XL-MS dataset appears sparse (derived from only 75 pmol of crosslinked material without SEC purification), which likely limits sensitivity and coverage. GPN1–3 appear inefficiently crosslinked (Extended Data Fig. 1e). The authors may consider: (i) repeating the XL-MS experiment starting from a larger amount of material and including a size-exclusion step prior to crosslinking; (ii) increasing crosslinker concentration to capture transient GPN interactions.

We thank the reviewer for the constructive criticism and suggestions. We would like to point out that (1) the crosslinking experiments described in our original manuscript (now referred to as dataset 1) were performed with the Size-exclusion chromatography (SEC)-purified complexes, and (2) in our experience, increasing the initial amounts of complexes beyond 50 pmol does not improve the depth of analysis (provided that the complexes are rather homogeneous) and elevated crosslinker concentrations often result in capturing excessive number of artificial crosslinks (e.g. due to partial aggregation during crosslinking reaction). For this reason, we have not used BS3 at concentration of >1.5 mM for crosslinking the RNA polymerase complexes. However, we now followed the suggestion of the reviewer and performed an XL-MS experiment using 100 pmol of cfPol II. Furthermore, we tried to increase the occupancy of GPN1 and GPN3 as well as GDOWN1 and RPAP2 on the cfPol2 by adding the respective recombinant proteins to the endogenous cfPol II. After re-purification by SEC, crosslinking with 2 mM BS3 was carried out (dataset 2). Although additional crosslinks of GPN1 and GPN3 were identified in the new dataset, we note that the observed overall modest increase of observed crosslink numbers resulted, on the other hand, in a drastic increase of overlength crosslinks (from approx. 10 to 28%). We have to admit that, regrettably, our cryoEM and XL-MS data are insufficient to model GPN1 and GNP3 into the 3D volume.

2. The presentation of the XL-MS data should also include a mapping of crosslinks to the resolved structure with range of Ca-Ca a histogram showing the crosslink distances observed with a violation rate to determine if both datasets are consistent. Additionally, it is unclear which FDR (1 or 5%) was used in the pLINK analysis of the XL-MS data. Please confirm which one was used for the dataset presented.

In the Supplementary Table 1 that contained XL-MS data, we specified which crosslinks were observed at spectral level FDR of 1 and 5%. We realized, however, that the submitted version of this table was apparently corrupted upon conversion into PDF. We apologize for this. We now provide this table in Excel format. We included additionally

the new analysis that was done with pLink3.0.17 and filtered at peptide-pair level FDR 1 and 5% for the original and newly acquired data (datasets 1 and 2, respectively). We also created distance distribution histograms that are shown in ED Figure 1e.

3. For the assessment of the CryoEM data, please include an FSC, Map vs. Model FSC, viewing angle distribution (or 3DFSC) and a figure showing the fitting of the model into the solved density. Also, in the extended data figure 2, please enlarge the 2D class averages as they are currently difficult to interpret.

We thank the reviewer for the feedback and we now include FSC plots, angular distribution, and model vs map plots for both cryo-EM maps (ED Figure 4). Further, we now include two ED Figure panels showing the initial AlphaFold 3 model with the cryo-EM density as well as the final model in the density (ED Figure 6e,d). Additionally, we now also include ED Figure panels showing the structural details of the GDOWN1-Pol II interactions including the EM density (ED Figure 7).

4. To demonstrate the steric mechanism of Gdown1 inhibition of transcription, the authors compare their structure to the TTF2-Pol II Alphafold3 prediction. Please include a pLDDT, iPTM and PTM scores to assess the quality of the prediction. The included PAE plot is difficult to understand, perhaps the inclusion of a zoomed panel or enlargement of the panel to allow for a clearer demonstration of the predicted interface?

We thank the reviewer for the suggestion and now added a zoomed-in view of the PAE plot for better visualization as well as added a PAE key for understanding (ED Figure 10). The pLDDT, iPTM and PTM scores for AF3 are reported in the legend of ED Figure 10.

Further to these points, additional minor points regarding the manuscript should be addressed:

5. The legend for figure 3b does not correspond to the figure.

We thank the reviewer for spotting this, we corrected it.

6. In figure 3d, the authors use a transcription activity assay to demonstrate the Gdown1 inhibition of Pol II transcription. However, for quantification authors should include a densitometric analysis across replicates with a statistical significance of comparison.

We thank the reviewer for this suggestion and now include quantifications for all transcription assays performed.

7. Extended Data Fig. 1b-c-d: some panels are missing molecular weight markers.

We thank the reviewer for spotting this, we corrected it.

8. For Extended Data Fig. 6f the boxes on the PAE plot are blue instead of red.

We thank the reviewer for spotting this, we corrected it (ED Figure 10).

Reviewer #4 (Remarks to the Author):

This manuscript by Schmitzova et al describes cryo-EM and mass spectrometry approaches to study the cytoplasmic form of RNA polymerase II (cfPolII) prior to assembly and import into the nucleus. The authors fractionated lysate from a human cell line to isolate a cytoplasmic complex using an affinity tag on the PolII subunit RPB1. In addition to cfPolII, further proteins co-purified, which have been implicated in PolII assembly (Gdown1, RPAP2, and the GPN1-GPN3 heterodimer). With respect to Gdown1, the manuscript comes to similar conclusions as a recent bioRxiv pre-print from the lab of Carrie Bernecky, which is reassuring. But here's where the story ends and to me the present manuscript feels very much like a preliminary draft and lacks the complementary biochemical data (and/or additional structural data) present in the bioRxiv preprint from the Bernecky lab. In the current form I am not in favor of publication.

We thank the reviewer for taking the time to review our manuscript. As outlined below (and above), we have added additional biochemical data to substantiate our conclusions and we believe they have improved the manuscript.

The abstract reads like a press release and not like a scientific abstract. I'd suggest using more neutral, objective language to let the data's significance speak for itself.

We thank the reviewer for this feedback. We have revised the text to reflect a neutral, data-centric narrative.

For example, how do the binding interfaces of Gdown1 and PolII indicate a stabilization of PolII assembly intermediates – the same interface was seen with fully assembled nuclear PolII and the authors themselves say they isolated fully assembled PolII (and not an intermediate) so we cannot know what the assembly intermediate may look like and this statement is a hyperbole.

We have now included experiments to test whether GDOWN1 is able to stabilize subassemblies of Pol II (See Reviewer-1, ED Figure 8a,b). In particular, we find that GDOWN1 stabilizes a subassembly comprising RPB3, RPB10, RPB11 and RPB12. Co-expression with GDOWN1 improves soluble expression and the stability of this sub-complex. Conversely, GDOWN1 does not promote the expression of RPB2-RPB9 subassembly and appears to negatively influence this process. Based on these results,

we have revised the text on how the presence of GDOWN1 might influence the association of the Pol II subcomplexes during biogenesis.

Overlap of Gdown1 and TFIIB and TFIIF have been reported before by the Roeder lab and others and it is not a new finding.

We thank the reviewer for pointing us to previous literature. Indeed, it was previously shown that GDOWN1 biochemically competes with TFIIB and TFIIF. However, the exact binding site for GDOWN1 on the Pol II surface was unknown. With our high-resolution structure, we now present the exact location of GDONW1 on the Pol II surface and can therefore explain the biochemical competition with these factors.

As far as I can see, there is no model to propose Gdown1's role in assembly, import, and transcription regulation – i.e. a model of Gdown1 implicated in these three independent processes – another exaggeration in my view.

(see our response below)

Is the clamp domain visible in low-pass filtered maps? Is it disordered or oscillating? In the latter case, have the authors tried focused 3D classification - please comment on this in the paper.

We thank the reviewer for the suggestion. We in fact tried this extensively during data processing, but the clamp domain remains invisible. We added a clarification for this in the text.

The authors show focused classification for the RPAP2 density (Extended Fig 3) but we learn next to nothing about it in the manuscript.

We agree with the reviewer that we do not learn much new about RPAP2 from our data. Nevertheless, with the aim to present the best possible molecular model for cfPol II, we used the focussed map for RPAP2 (Map B) to build the RPAP2 model with the highest possible quality. We therefore will keep this ED Figure as part of the manuscript. As shown in ED Figure 5, RPAP2 in our structure indeed does not deviate much from previously known structures and we therefore do not further comment on it in the text.

Page 4, line 33 (and page 5, line 27): "…first atomic model of Gdown1" feels like hyperbole – about a third of the protein could be modeled.

We respectfully disagree with the reviewer here. Previous models of GDOWN1 were limited to low resolution and therefore only allowed a global docking of GDOWN1 fragments on the Pol II surface. On the contrary, our structure for the first time allows to build an atomic model for the functionally important parts of GDOWN1 that interact with and regulate Pol II. Furthermore, we tend to not align with the suggestion that a model should only be considered atomic if the complete protein chain is resolved, as

this is rarely achievable in practice. We therefore decided to keep the statement as it is.

Page 6, line17-19: This sentence does not make any sense.

We apologize that this incomplete sentence remained in the manuscript. We have fixed this in the revised text.

The model presented in the Discussion is highly speculative and almost exclusively based on data published in other studies.

We have added additional biochemical data to support our model. The revised manuscript includes:

- co-expression experiments of Pol II subassemblies in the absence or presence of GDOWN1 and demonstrate that GDOWN1 influences the solubility and stability of the RPB3, RPB10, RPB11 and RPB12 subassembly (ED Figure 8a,b)
- biochemical data showing that GDOWN1 is the main reason why cfPol II is inactive in transcription initiation, as compared to RPAP2 (Figure 4d,e)
- phosphormimetic mutational analysis of GDOWN1 at position S270 showing the (partial?) release of the repression of transcription initiation in a highly purified in vitro system (Figure 4d,e)

These results support our model that: 1) GDOWN1 binds Pol II prior to its assembly stabilises this process and participates in the nuclear import. 2) The imported cfPol II is transcriptionally repressed and this inhibition needs to be released through GDOWN1 phosphorylation.

While we also cite published data to put our hypotheses forward, it is our structural and biochemical data that strengthen these conclusions. We are therefore are confident that our manuscript provides a conceptual advance beyond prior data.

Fig. 3: The legend does not match the figure. There's no marker in the left panel of 3c

We thank the reviewer for spotting this, we corrected it (now in Figure 4).

Extended Data Fig. 6d and 6f: Colors described in legend do not match figure.

We thank the reviewer for spotting this, we corrected it (now in ED Figure 10).

Extended Data Table 2: initial particle images number is too low. Are there really 0% Rotamer outliers?

We thank the reviewer for spotting this, we corrected the particle number reported. We also confirm that the structure has 0% rotamer outliers.

Please provide FSC plots for the final reconstructions.
We have added the FSC plots in ED Figure 4.

Response to Reviewer #1:

Remaining comments:
Our responses are shown in blue.

Fig. 4b (figure legend): Specify whether triplicate experiments were performed as biological or technical replicates.
We thank the reviewer for spotting this, we used technical replicates. We corrected this.

Extended Data Fig. 7: The authors should consider showing all side chains, rather than a select few, to support their register and contact surface assignment. Are all the side chains implicated in polar interactions actually within distances suitable for hydrogen bonding or formation of salt bridges?

We thank the reviewer for spotting this, we show now all side chains in Supplementary Figure 7. We also checked all the side chains implicated in polar interactions and they are within 3.0-3.9 Å. Given the local resolution of ~3-4 Å, these distances fall within the range suitable for hydrogen bonding or formation of salt bridges.

Extended Data Fig. 8: Why did the authors elect to swap the position of the tag for their co-purification assay with/without GDOWN1? The position of the tag alone could affect protein stability or complex formation.

We thank the reviewer for this question. We decided for this strategy to confirm that GDOWN1 specifically binds sub-complexes of Pol II. We agree that the tag on Pol II subunits could affect the protein stability. After swapping the tag from RPB2/3 to GDOWN1, both Pol II subunits still express in a soluble manner, indicating that the tag removal does not interfere with the intended experiment. Further, the MBP tag could influence formation of Pol II subcomplexes. However, the MPB tag was added to the N-terminus of RPB2/3 proteins using a long linker, which should not prevent proper folding and potential interactions with other Pol II subunits.

Data integrity: Extended Data Fig. 8 contains multiple instances of apparent lane splicing that are not indicated. If this observation is accurate, this must be corrected.

We thank the reviewer for spotting this, we corrected it.
